# (Pre)diabetes and a higher level of glycaemic measures are continuously associated with corneal neurodegeneration assessed by corneal confocal microscopy: the Maastricht Study

**DOI:** 10.1007/s00125-023-05986-5

**Published:** 2023-08-17

**Authors:** Sara B. A. Mokhtar, Frank C. T. van der Heide, Karel A. M. Oyaert, Carla J. H. van der Kallen, Tos T. J. M. Berendschot, Fabio Scarpa, Alessia Colonna, Bastiaan E. de Galan, Marleen M. J. van Greevenbroek, Pieter C. Dagnelie, Casper G. Schalkwijk, Rudy M. M. A. Nuijts, Nicolaas C. Schaper, Abraham A. Kroon, Miranda T. Schram, Carroll A. B. Webers, Coen D. A. Stehouwer

**Affiliations:** 1 CARIM School for Cardiovascular Diseases, Maastricht University, Maastricht, the Netherlands; 2 Department of Internal Medicine, Maastricht University Medical Center, Maastricht, the Netherlands; 3 School of Mental Health and Neuroscience, Maastricht University, Maastricht, the Netherlands; 4 University Eye Clinic Maastricht, Maastricht University Medical Center, Maastricht, the Netherlands; 5 Department of Information Engineering, University of Padova, Padova, Italy; 6 Department of Internal Medicine, Radboud University Medical Center, Nijmegen, the Netherlands; 7 Department of Internal Medicine, Division of Endocrinology and Metabolic Disease, Maastricht University Medical Center, Maastricht, the Netherlands; 8 Care and Public Health Research Institute, Maastricht University, Maastricht, the Netherlands; 9 Heart and Vascular Center, Maastricht University Medical Center, Maastricht, the Netherlands

**Keywords:** Corneal neurodegeneration, Glycaemic measures, (Pre)diabetes

## Abstract

**Aims/hypothesis:**

To assess the associations between glucose metabolism status and a range of continuous measures of glycaemia with corneal nerve fibre measures, as assessed using corneal confocal microscopy.

**Methods:**

We used population-based observational cross-sectional data from the Maastricht Study of *N*=3471 participants (mean age 59.4 years, 48.4% men, 14.7% with prediabetes, 21.0% with type 2 diabetes) to study the associations, after adjustment for demographic, cardiovascular risk and lifestyle factors, between glucose metabolism status (prediabetes and type 2 diabetes vs normal glucose metabolism) plus measures of glycaemia (fasting plasma glucose, 2 h post-load glucose, HbA_1c_, skin autofluorescence [SAF] and duration of diabetes) and composite *Z*-scores of corneal nerve fibre measures or individual corneal nerve fibre measures (corneal nerve bifurcation density, corneal nerve density, corneal nerve length and fractal dimension). We used linear regression analysis, and, for glucose metabolism status, performed a linear trend analysis.

**Results:**

After full adjustment, a more adverse glucose metabolism status was associated with a lower composite *Z*-score for corneal nerve fibre measures (*β* coefficients [95% CI], prediabetes vs normal glucose metabolism −0.08 [−0.17, 0.03], type 2 diabetes vs normal glucose metabolism −0.14 [−0.25, −0.04]; linear trend analysis showed a *p* value of 0.001), and higher levels of measures of glycaemia (fasting plasma glucose, 2 h post-load glucose, HbA_1c_, SAF and duration of diabetes) were all significantly associated with a lower composite *Z*-score for corneal nerve fibre measures (per SD: −0.09 [−0.13, −0.05], −0.07 [−0.11, −0.03], −0.08 [−0.11, −0.04], −0.05 [−0.08, −0.01], −0.09 [−0.17, −0.001], respectively). In general, directionally similar associations were observed for individual corneal nerve fibre measures.

**Conclusions/interpretation:**

To our knowledge, this is the first population-based study to show that a more adverse glucose metabolism status and higher levels of glycaemic measures were all linearly associated with corneal neurodegeneration after adjustment for an extensive set of potential confounders. Our results indicate that glycaemia-associated corneal neurodegeneration is a continuous process that starts before the onset of type 2 diabetes. Further research is needed to investigate whether early reduction of hyperglycaemia can prevent corneal neurodegeneration.

**Graphical Abstract:**

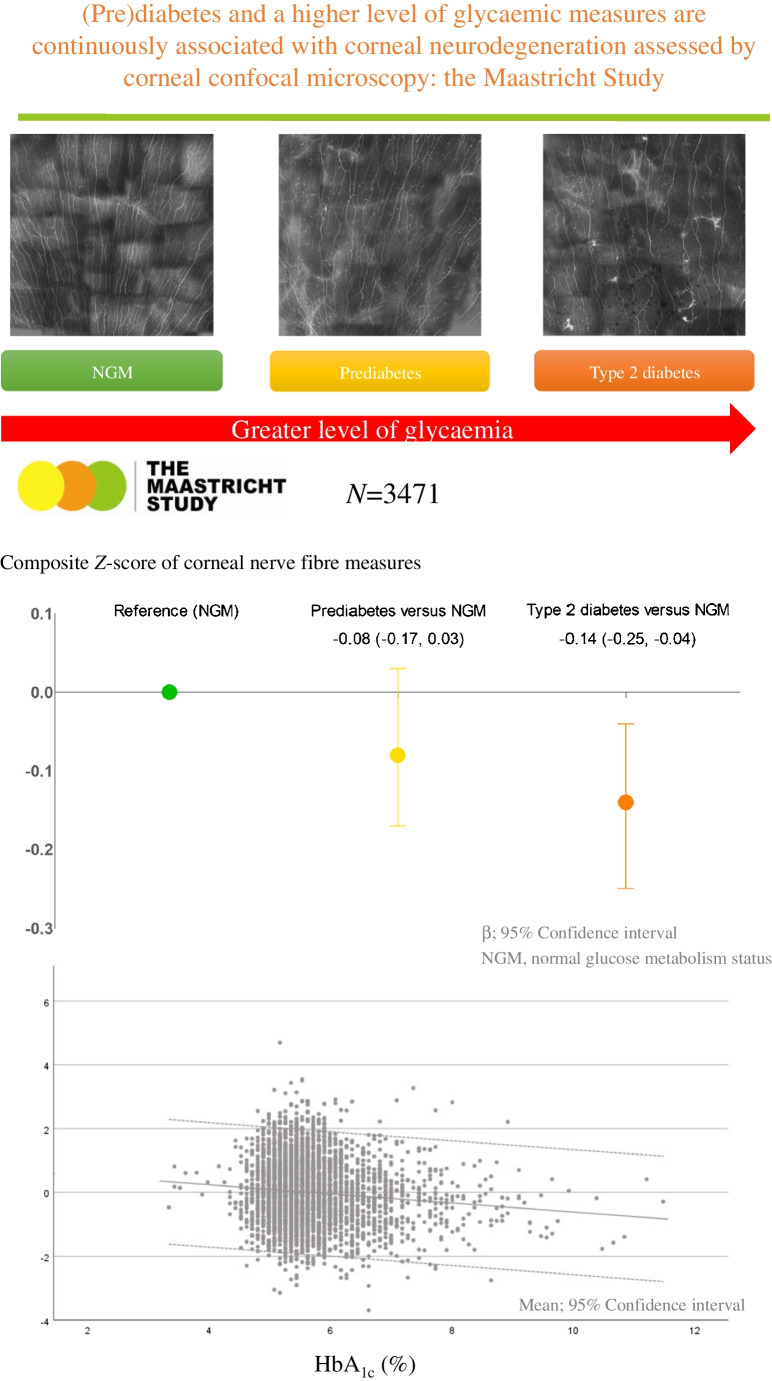

**Supplementary Information:**

The online version contains supplementary material available at 10.1007/s00125-023-05986-5.


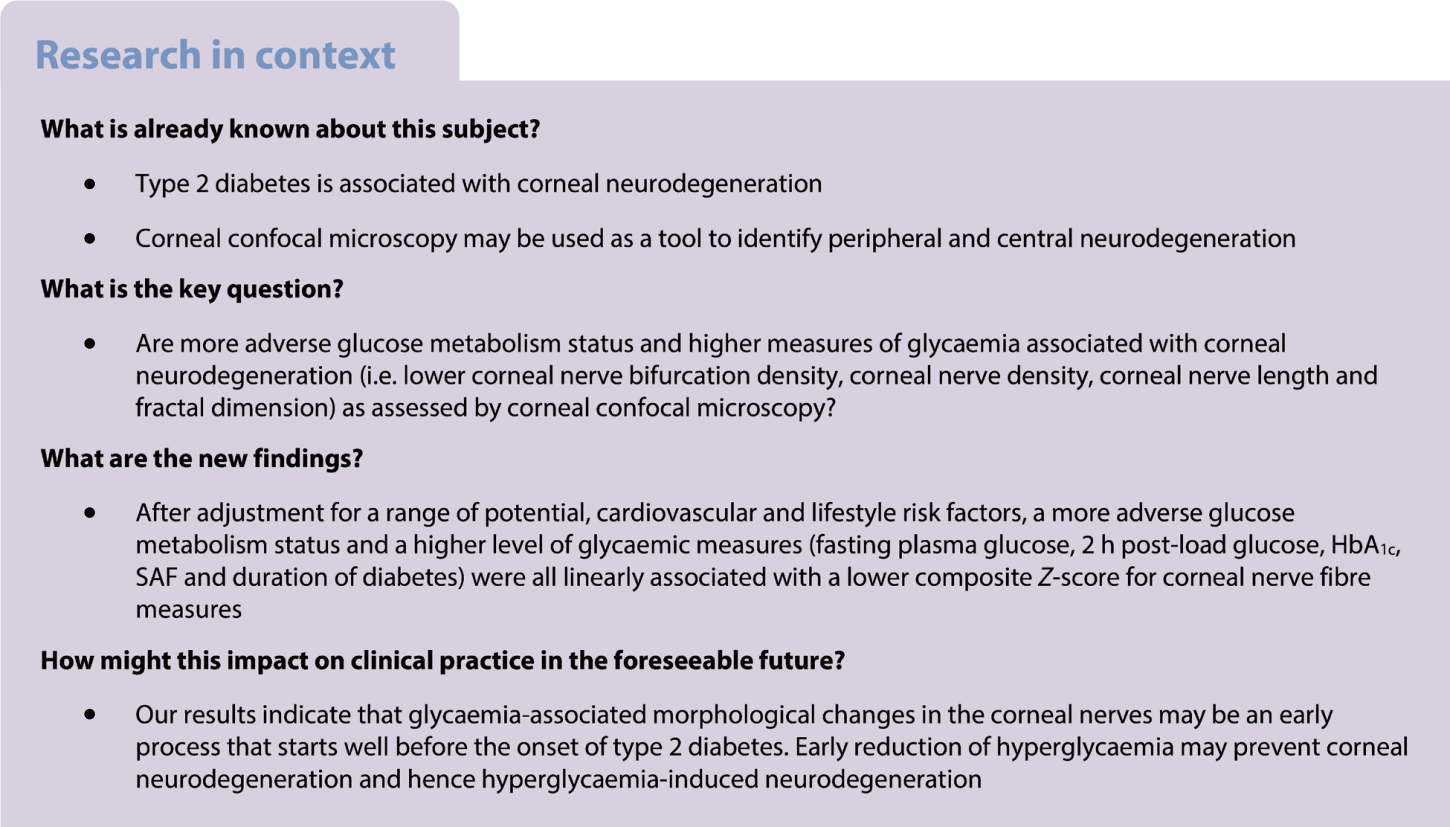


## Introduction

Diabetes can lead to a vast spectrum of systemic complications including central and peripheral neurodegeneration [1, 2]. Techniques that allow early detection and regular follow-up of hyperglycaemia-induced neurodegeneration are thus of major importance.

As postulated in the ‘ticking clock hypothesis’, hyperglycaemia-mediated damage is a continuous (i.e. linear) process that starts before the onset of type 2 diabetes [3, 4]. Indeed, we have previously demonstrated linear associations of adverse glucose metabolism status and higher levels of glycaemic measures with lower retinal nerve fibre layer thickness [5], more brain structural abnormalities [6, 7], worse peripheral nerve function [8] and lower heart rate variability [9], which are all measures of neurodegeneration and/or neural dysfunction. Additionally, chronic hyperglycaemia accelerates the formation of advanced glycation end-products (AGEs), which are considered a major initiator of neurodegeneration [10]. AGEs are protein-bound compounds that exhibit intrinsic fluorescence properties, which can be measured non-invasively as skin autofluorescence (SAF) [11]. We have shown previously that AGEs assessed as SAF are associated with lower retinal nerve fibre layer thickness [5].

Various instruments and techniques are currently available to allow scientists to monitor central and peripheral nervous system activity. However, these methods are often time-consuming and have relatively high costs [12, 13], making them impractical for routine and large-scale use. There thus is a need for a sensitive and practical method that allows detection of early hyperglycaemia-mediated neurodegeneration.

Here we focus on hyperglycaemia as a determinant of morphological changes in small nerve fibres (unmyelinated C-fibres) assessed by corneal confocal microscopy, an in vivo, non-invasive and sensitive measure [14] to assess corneal nerve degeneration. The current literature supports the concept that hyperglycaemia affects corneal nerve fibres [15], and that corneal confocal microscopy may be used as a tool to identify peripheral [15, 16] and central neurodegeneration [16]. Moreover, studies have suggested that alterations in corneal nerves morphology may serve as an early indicator of diabetic neuropathy, offering a potential opportunity for timely diagnosis and treatment [17, 18]. However, these studies had several important limitations. Specifically, previous studies were not population-based, did not adjust for cardiovascular risk and lifestyle factors, and did not investigate whether various measures of glycaemia are linearly associated with measures of corneal nerve fibre damage.

Hence, we investigated, in a large, well-characterised population-based cohort study, whether a more adverse glucose metabolism status and higher measures of glycaemia are associated with neurodegeneration assessed using measures of corneal nerve fibre damage, i.e. lower corneal nerve bifurcation density, corneal nerve density, corneal nerve length and fractal dimension, as measured by corneal confocal microscopy.

## Methods

### Study population and design

We used data from the Maastricht Study, an observational prospective population-based cohort study enriched with type 2 diabetes individuals. Individuals with type 2 diabetes were over-sampled for efficiency reasons, i.e. to increase the statistical power to identify any potential contrasts between individuals with and without type 2 diabetes. The rationale and methodology have been described previously [19]. In brief, the study focuses on the aetiology, pathophysiology, complications and comorbidities of type 2 diabetes mellitus, and is characterised by an extensive phenotyping approach. Eligible for participation were all individuals aged between 40 and 75 years living in the southern part of the Netherlands. Participants were recruited through mass media campaigns and from the municipal registries and the regional Diabetes Patient Registry via mailings. Recruitment was stratified according to known type 2 diabetes status [19]. The present report includes cross-sectional data for *N*=3471 participants who completed the baseline survey between November 2010 and December 2017. The examinations for each participant were performed within a time window of 3 months. Corneal confocal microscopy measurements were performed between April 2013 and January 2017. Individuals who participated in the Maastricht Study before the start of corneal confocal microscopy measurement (i.e. before April 2013) were re-invited (*n*=974) (‘catch-up visit’). For these participants, there was a median time interval (‘visit interval or lag time’) of 5.2 years between corneal confocal microscopy measurements and all other measurements. The study has been approved by the institutional medical ethical committee (NL31329.068.10) and the Minister of Health, Welfare and Sports of the Netherlands (permit 131088–105234-PG). All participants gave written informed consent [19].

### Assessment of corneal confocal microscopy measurements (outcome)

We used corneal confocal microscopy using a Heidelberg retina tomograph III (HRT3), Rostock cornea module (Heidelberg Engineering, Germany) to image corneal nerves [14] of the left eye only for logistical reasons. Before the measurement, we anaesthetised both eyes using oxybuprocaine hydrochloride 0.4 mg/ml eye drops (Minims; Bausch & Lomb, France) to eliminate all causes of blinking, and wetted both eyes using carbomer 2 mg/g eye gel (Vidsic Bausch & Lomb) to ensure optimal contact between the cornea and the applanation cap. Further methodological details are provided in the electronic supplementary material (ESM) Methods.

Individuals who had a corneal transplant or had a corneal infection of the left eye were excluded from the measurements. Participants were instructed to fix their vision on a white light throughout the scan. Trained research assistants imaged the sub-basal nerve plexus layer in the central part of the cornea according to a standard operating procedure. We assessed multiple recordings of 400×400 μm (384×384 pixels, 8-bit) assembled using a composite algorithm implemented in the HRT3 user interface (Heidelberg Engineering), as previously described [14]. Real-time mapping was performed on an area up to 1600×1600 µm (1536×1536 pixels, 8-bit) that partially included the inferior whorl in some of the composite images. No measures were taken to include or exclude this region. The advantage of performing large scale corneal confocal microscopy imaging (1600 × 1600 μm) is described in the ESM Methods.

We used the U-net-based convolutional neural network to fully automatically trace the corneal nerves [20] (ESM Fig. 1) and analysed the following indices: corneal nerve bifurcation density (number of bifurcation points [branching points] per mm^2^), corneal nerve density (total number of corneal nerve fibres [including both main fibres and branches] per mm^2^, with ‘main nerve fibres’ referring to the largest and most prominent nerve fibres), corneal nerve length (total length of corneal nerve fibres in mm, both main and branches, per mm^2^) and corneal nerve fractal dimension (quantification of the nerve structure complexity). Composite segmented images were reviewed manually, and images were considered to be usable (*n*=3623) if 50% or more of the total captured area was of good quality (see ESM Methods for more details). ESM Fig. 2 provides examples of included and excluded images. We also assessed the location of the captured images based on the orientation of the corneal nerves (ESM Fig. 3). The intra-class and inter-observer correlation coefficients, both indices of reliability, were ≥ 0.97 and ≥ 0.89, respectively [21].

### Assessment of determinants

#### Glucose metabolism status

After an overnight fast, participants underwent a standardised seven-point OGTT, as part of which venous samples were collected at 15, 30, 45, 60, 90 and 120 min post-ingestion of a 75 g glucose drink. All participants underwent an OGTT except those who used insulin or had a fasting plasma glucose concentration above 11.0 mmol/l. Based on fasting plasma glucose and 2 h post-load glucose, glucose metabolism status was determined as normal glucose metabolism, prediabetes (defined as impaired fasting glucose, impaired glucose tolerance or both), type 2 diabetes or other types of diabetes (including type 1 diabetes) according to the WHO 2006 criteria [22]

#### Measures of glycaemia

Fasting plasma glucose (mmol/l) and HbA_1c_ (mmol/mol, %) were determined in venous plasma samples collected after an overnight fast. The value for 2 h post-load glucose (mmol/l) was determined in venous plasma collected at 120 min after ingestion of the glucose drink. The levels of AGEs in the skin were assessed using an AGE Reader (DiagnOptics Technologies, the Netherlands). In brief, the AGE Reader is a desktop device that uses the characteristic fluorescent properties of certain AGEs to quantify their accumulation in the skin as SAF (arbitrary units) [23]. The AGE Reader illuminates a skin surface of 4 cm^2^, shielded from other light, and uses the ratio of the reflection of fluorescent light (wavelength 420–600 nm) to non-fluorescent light (300–420 nm) to calculate SAF (see ESM Methods for more details). The duration of diabetes in individuals with type 2 diabetes was assessed using a questionnaire.

### Covariates

As described previously [19], we assessed educational level (low, intermediate, high), socioeconomic status (income level and occupational status) [24], smoking status (never, former, current), alcohol use (none, low, high), history of ocular disorders (corneal diseases or uveitis), and use of contact lenses by questionnaire, assessed the presence of retinopathy in both eyes via fundus photography (see ESM Methods for more details), assessed dietary habits (‘dietary intake’) using the Dutch Healthy Diet index sum score, which is a measure of adherence to the Dutch dietary guidelines 2015 (see ESM Methods for more details) [25], based on a validated food frequency questionnaire [26], assessed use of lipid-modifying, antihypertensive and glucose-lowering medication as part of a medication interview, assessed weight (kg), height (cm) and waist circumference (cm) during a physical examination, calculated BMI (kg/m^2^) based on body weight and height, measured office and 24 h ambulatory BP (mmHg), measured total daily physical activity (h/day) using an accelerometer [27], measured lipid profile and plasma biomarkers of low-grade inflammation [28] (i.e. high-sensitive C-reactive protein, serum amyloid A, IL-6, IL-8 and TNF-α) in fasting venous blood samples, measured urinary albumin excretion in two 24 h urine collections, calculated the eGFR based on serum creatinine only, as values for cystatin C were not available for all study participants, and assessed sensory neuropathy as having neuropathic pain using the Dutch version of the DN4 interview [29], via impaired uni- or bilateral vibration perception, measured three times using a Horwell neurothesiometer (Scientific Laboratory Supplies, UK) at the distal phalanx of the hallux of the right and left foot [19], and/or on the basis of use of medication prescribed for neuropathic pain (gabapentin, pregabalin, duloxetine, amitriptyline, nortriptyline or carbamazepine, the latter only in individuals without a diagnosis of epilepsy).

### Statistical analyses

Population characteristics and measures of the corneal nerves were described for the total study population and by tertiles of unweighted composite *Z*-score for corneal nerve fibre measures using the appropriate descriptive statistics (Table 1). Before we calculated the composite *Z*-score, we checked whether associations of potential determinants with the individual corneal nerve fibre measures were not directionally different, and this was the case. Additional general study population characteristics and general study population characteristics of the included and excluded participants are provided in ESM Table 1 and ESM Table 2. All analyses were performed using Statistical Package for Social Sciences version 25.0 (IBM SPSS, USA). For all analyses, a *p* value < 0.05 was considered statistically significant.

**Table 1:** General study population characteristics according to tertiles of composite *Z*-score for corneal nerve fibre measures in the study population with complete data on glucose metabolism status

		Composite *Z*-score for corneal nerve fibre measures		
Characteristic	Total study population (*n*=3471)	Tertile 1 (high) (*n*=1157)	Tertile 2 (middle) (*n*=1157)	Tertile 3 (low) (*n*=1157)
Age (years)	59.4±8.7	58.4±8.7	59.4±8.6	60.6±8.6
Men	1681 (48.4)	490 (42.4)	579 (50.0)	612 (52.9)
Educational level				
High	1332 (38.4)	440 (38.0)	441 (38.1)	451 (39.0)
Medium	956 (27.5)	336 (29.0)	324 (28.0)	296 (25.6)
Low	1183 (34.1)	381 (32.9)	392 (33.9)	410 (35.4)
Glucose metabolism status				
Normal glucose metabolism	2225 (64.1)	799 (69.1)	742 (64.1)	684 (59.1)
Prediabetes	509 (14.7)	164 (14.2)	165 (14.3)	180 (15.6)
Type 2 diabetes	729 (21.0)	193 (16.7)	246 (21.3)	290 (25.1)
Other type of diabetes	8 (0.2)	1 (0.1)	4 (0.3)	3 (0.3)
Measures of glycaemia				
Fasting plasma glucose (mmol/l)	5.7±1.4	5.5±1.1	5.8±1.5	5.9±1.5
2 h post-load glucose (mmol/l)	6.0 (4.9–8.3)	5.9 (4.8–7.9)	6.0 (4.9–8.3)	6.3 (5.0–9.0)
HbA_1c_ (mmol/mol)	38.6±8.4	37.4±6.8	38.8±9.0	39.5±9.1
HbA_1c_ (%)	5.7±0.8	5.6±0.6	5.7±0.8	5.8±0.8
SAF (AU)	2.1±0.5	2.1±0.4	2.1±0.5	2.2±0.5
Duration of type 2 diabetes (years)	3.0 (0.0–8.3)	2.0 (0.0–7.0)	3.0 (0.0–9.0)	4.0 (0.3–9.0)
Waist circumference (cm)	94.3±13.2	92.8±12.6	94.8±13.2	95.4±13.2
Total to HDL-cholesterol ratio	3.4 (2.8–4.2)	3.3 (2.7–4.2)	3.4 (2.8–4.2)	3.4 (2.8–4.2)
Use of lipid-modifying medication	1009 (29.1)	312 (27.0)	327 (28.3)	370 (32.0)
Office systolic BP (mmHg)	132.8±17.6	130.8±16.9	133.4±17.7	134.2±17.9
Office diastolic BP (mmHg)	75.5±9.9	74.9±9.7	75.3±9.9	75.6±10.0
Use of antihypertensive medication	1220 (35.1)	372 (32.2)	403 (34.8)	445 (38.5)
Smoking status				
Never	1341 (38.6)	474 (41.0)	472 (40.8)	395 (34.1)
Former	1711 (49.3)	540 (46.7)	566 (48.9)	605 (52.3)
Current	419 (12.1)	143 (12.4)	119 (10.3)	157 (13.6)
Alcohol consumption				
None	632 (18.2)	219 (18.9)	203 (17.5)	210 (18.2)
Low	2077 (59.8)	697 (60.2)	698 (60.3)	682 (58.9)
High	762 (22.0)	241 (20.8)	256 (22.1)	265 (22.9)
Sensory neuropathy^a^, yes vs. no	520 (15.1)	142 (12.4)	184 (16.1)	194 (16.9)
Neuropathic pain^b^	308 (8.9)	94 (8.2)	108 (9.4)	106 (9.2)
Impaired unilateral vibration perception	244 (7.0)	51 (4.4)	89 (7.7)	104 (9.0)
Impaired bilateral vibration perception^c^	100 (3.1)	18 (1.6)	41 (3.8)	41 (3.9)
Mean neurothesiometer outcome (V)^d^	10 (6.8–15.6)	9.4 (6.4–14.5)	10.0 (7.0–15.7)	10.8 (7.2–17.3)
No corneal confocal microscopy scan at baseline	974 (28.1)	274 (23.7)	293 (25.3)	407 (35.2)
Corneal confocal microscopy interval (years)	5.2 (4.9–5.8)	5.1 (4.8–5.5)	5.1 (4.8–5.8)	5.2 (5.0–5.8)
Corneal nerve bifurcation density	73.9±39.9	118.1±31.4	67.4±13.9	36.3±13.9
Corneal nerve density	79.6±24.3	105.0±17.1	77.7±10.7	56.0±12.6
Corneal nerve length	14.9±4.4	19.6±2.5	14.8±1.6	10.3±2.3
Corneal nerve fractal dimension	1.3±0.1	1.4±0.0	1.4±0.1	1.2±0.1

Data are presented as means ± SD, median (IQR) or *n* (%)

^a^Sensory neuropathy was defined as having neuropathic pain, impaired uni- or bilateral vibration perception, and/or use of medication prescribed for neuropathic pain (gabapentin, pregabalin, duloxetine, amitriptyline, nortriptyline or carbamazepine, the latter only in individuals without a diagnosis of epilepsy). In the analysis of sensory neuropathy, it is important to note that there were missing data. The sample size for total study population *n*=3439, for each tertile is as follows: Tertile 1 (high) *n*=1146, Tertile 2 (middle) *n*=1146 and Tertile 3 (low) *n*=1147

^b^In the analysis of neuropathic pain, it is important to note that there were missing data. The sample size for total study population *n*=3450, for each tertile is as follows: Tertile 1 (high) *n*=1149, Tertile 2 (middle) *n*=1151 and Tertile 3 (low) *n*=1150

^c^In the analysis of impaired bilateral vibration perception it is important to note that there were missing data. The sample size for total study population *n*=3256, for each tertile is as follows: Tertile 1 (high) *n*=1108, Tertile 2 (middle) *n*=1092 and Tertile 3 (low) *n*=1056

^d^On right and left first toe

AU, arbitrary units

#### Main analyses

We used multivariable linear regression analyses to investigate the associations of determinants (i.e. glucose metabolism status [entered as dummy variables for prediabetes, type 2 diabetes, or other types of diabetes vs normal glucose metabolism] and measures of glycaemia [standardised fasting plasma glucose, 2 h post-load glucose, HbA_1c_, SAF and duration of diabetes]) with outcomes (i.e. composite *Z*-score for corneal nerve fibre measures [main outcome] and standardised corneal nerve fibre measures [i.e. corneal nerve bifurcation density, corneal nerve density, corneal nerve length and corneal nerve fractal dimension]).

We performed an analysis of the linear trend for the association with glucose metabolism status as a determinant. To estimate the linear trend, we entered glucose metabolism status into the model as an ordinal variable (i.e. glucose metabolism status was coded as normal glucose metabolism = 0, prediabetes = 1, type 2 diabetes = 2). In a *p* for trend analysis, we excluded participants with other types of diabetes (such as type 1 diabetes) because other types of diabetes do not constitute part of the spectrum of deterioration of glucose metabolism status from normal glucose metabolism to prediabetes and type 2 diabetes. Then, we checked whether we could assume a linear trend by comparing the statistical variance explained by the statistical model in which glucose metabolism status was entered as dummy variables to that obtained using the statistical model in which glucose metabolism status was entered as an ordinal variable. We used a likelihood ratio test to assess whether the difference in the amount of variance explained by both models was statistically significant. A *p* value > 0.05 indicates that both models are not different, and thus a linear trend can be assumed.

We used several sets of adjustments. Model 1 shows crude results adjusted for corneal confocal microscopy lag time. Model 2, in addition to the adjustment in model 1, was adjusted for age, sex and educational status (high, medium, low). We chose these variables because they are key potential confounders. Model 3, in addition to the adjustments in model 2, was adjusted for office systolic BP, use of antihypertensive medication (yes/no), waist circumference, total cholesterol to HDL-cholesterol ratio, use of lipid-modifying medication (yes/no), smoking status (never, former, current) and alcohol consumption status (none, low [for women, ≤7 alcohol units per day; for men, ≤14 alcohol units per day], high [for women, >7 alcohol units per day; for men, >14 alcohol units per day]). Biologically, these risk factors may be associated with hyperglycaemia and corneal neurodegeneration. All associations are expressed as standardised regression coefficients and the corresponding 95% CI. To facilitate interpretation, we show unstandardised regression coefficients (*β*) in ESM Table 3. The associations between glucose metabolism status and measures of glycaemia with corneal nerve fractal dimension had long strings of digits, near to zero, that we multiplied by 100 for convenient expression (in ESM Table 3).

We also analysed potential interaction with sex by adding an interaction term (i.e. potential determinant × sex) [30] to model 3 (ESM Table 4).

#### Additional analyses

To ensure the robustness of our findings, we performed several additional analyses as described in ESM Methods.

## Results

### Selection and characteristics of the study population

**Fig. 1:**
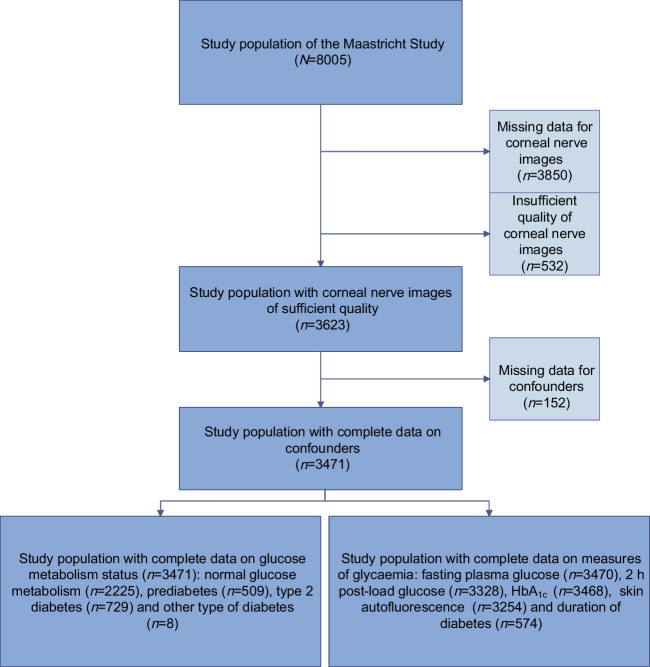
Selection of participants for inclusion in analyses

Figure 1 shows an overview of selection of the study population.

Table 1 and ESM Table 1 show the general participant characteristics according to tertiles of composite *Z*-score for corneal nerve fibre measures. Overall, participants with a lower composite *Z*-score for corneal nerve fibre measures were older and had a more adverse cardiovascular risk profile. The general characteristics of participants included in the study were comparable to those with missing data (ESM Table 2).

### Glucose metabolism status and corneal nerve fibre measures

**Fig. 2:**
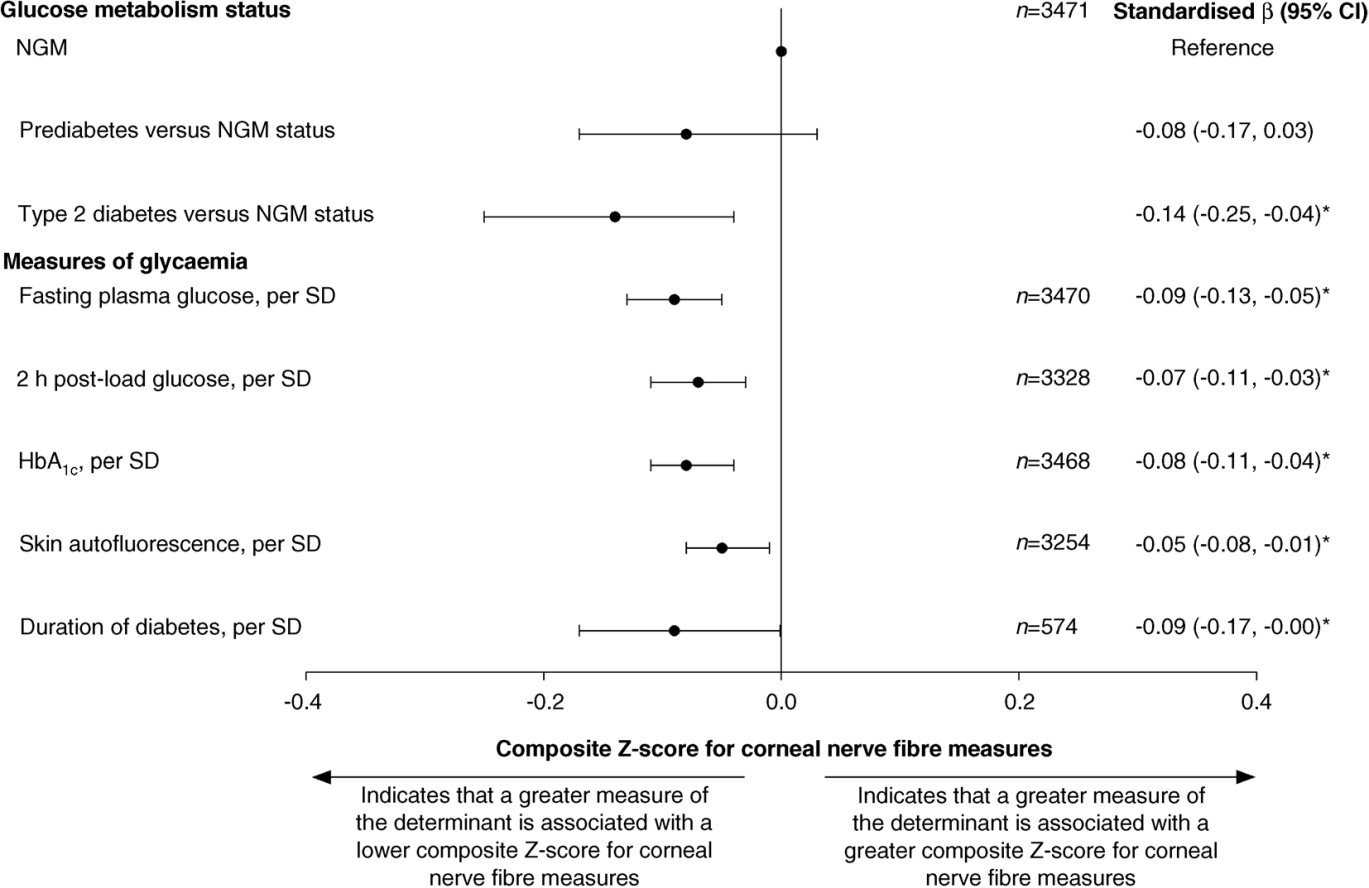
Associations between glucose metabolism status and measures of glycaemia with the composite *Z*-score for corneal nerve fibre measures. Standardised regression coefficients (*β*) represent the differences in corneal nerve fibre measures in SD, for prediabetes or type 2 diabetes vs NGM, or per SD greater for measures of glycaemia. For the glucose metabolism status, fasting plasma glucose, 2 h post-load glucose, HbA_1c_, SAF and duration of diabetes (for type 2 diabetes only) study populations, 1 SD corresponds to 0.9 (unit-less) for the composite *Z*-score for corneal nerve fibre measures, 1.4 mmol/l for fasting plasma glucose, 3.9 mmol/l for 2 h post-load glucose, 0.8% or 8.4 mmol/mol for HbA_1c_, 0.5 arbitrary units for SAF and 7.4 years for duration of diabetes. Variables entered into the models in addition to glucose metabolism status or measures of glycaemia were corneal confocal microscopy visit interval, age, sex educational status (high, medium, low), office systolic BP, total cholesterol to HDL-cholesterol ratio, use of antihypertensive or lipid-modifying medication (yes/no), waist circumference, smoking status (never, former, current) and alcohol consumption status (none, low, high). Asterisks indicate values that are statistically significant (*p*<0.05). NGM, Normal glucose metabolism

According to model 3, a more adverse glucose metabolism status was associated with a lower composite *Z*-score for corneal nerve fibre measures (*β* [95% CI]: type 2 diabetes vs normal glucose metabolism −0.14 [−0.25, −0.04], prediabetes vs normal glucose metabolism −0.08 [−0.17, 0.03]; linear trend analysis showed a *p* value of 0.001) (Table 2 and Fig. 2). In general, directionally similar associations were observed for individual corneal nerve fibre measures (Table 2).

**Table 2:** Associations of glucose metabolism status and measures of glycaemia with corneal nerve fibre measures

Determinant	Number of participants	Model	Composite *Z*-score for corneal nerve fibre measures	Corneal nerve bifurcation density	Corneal nerve density	Corneal nerve length	Corneal nerve fractal dimension
Glucose metabolism status	3471						
Prediabetes vs normal glucose metabolism		1	−0.13 (−0.22, −0.03)*	−0.09 (−0.19, 0.01)	−0.12 (−0.22, −0.03)*	−0.13 (−0.23, −0.04)*	−0.12 (−0.21, −0.02)*
		2	−0.08 (−0.17, 0.02)	−0.06 (−0.15, 0.04)	−0.08 (−0.18, 0.02)	−0.07 (−0.17, 0.02)	−0.07 (−0.17, 0.03)
		3	−0.08 (−0.17, 0.03)	−0.07 (−0.17, 0.03)	−0.09 (−0.19, 0.01)	−0.07 (−0.17, 0.03)	−0.05 (−0.15, 0.05)
Type 2 diabetes vs normal glucose metabolism		1	−0.21 (−0.30, −0.13)*	−0.13 (−0.21, −0.05)*	−0.16 (−0.25, −0.08)*	−0.22 (−0.30, −0.13)*	−0.28 (−0.36, −0.19)*
		2	−0.14 (−0.23, −0.05)*	−0.08 (−0.16, 0.01)	−0.10 (−0.18, −0.01)*	−0.13 (−0.22, −0.05)*	−0.21 (−0.30, −0.12)*
		3	−0.14 (−0.25, −0.04)*	−0.11 (−0.21, −0.001)*	−0.13 (−0.24, −0.03)*	−0.12 (−0.23, −0.02)*	−0.17 (−0.27, −0.06)*
Measures of glycaemia							
Fasting plasma glucose, per SD	3470	1	−0.11 (−0.14, −0.08)*	−0.08 (−0.12, −0.05)*	−0.09 (−0.12, −0.05)*	−0.11 (−0.14, −0.08)*	−0.12 (−0.16, −0.09)*
		2	−0.08 (−0.12, −0.05)*	−0.06 (−0.10, −0.03)*	−0.06 (−0.10, −0.03)*	−0.08 (−0.11, −0.04)*	−0.10 (−0.13, −0.06)*
		3	−0.09 (−0.13, −0.05)*	−0.08 (−0.12, −0.04)*	−0.08 (−0.12, −0.04)*	−0.08 (−0.12, −0.04)*	−0.08 (−0.12, −0.05)*
2 h post-load glucose, per SD	3328	1	−0.09 (−0.12, −0.06)*	−0.06 (−0.09, −0.03)*	−0.07 (−0.10, −0.04)*	−0.09 (−0.13, −0.06)*	−0.11 (−0.14, −0.07)*
		2	−0.06 (−0.10, −0.03)*	−0.04 (−0.08, −0.01)*	−0.05 (−0.08, −0.01)*	−0.06 (−0.10, −0.03)*	−0.08 (−0.12, −0.05)*
		3	−0.07 (−0.11, −0.03)*	−0.06 (−0.10, −0.02)*	−0.07 (−0.11, −0.03)*	−0.07 (−0.11, −0.03)*	−0.07 (−0.11, −0.03)*
HbA_1c_, per SD	3468	1	−0.10 (−0.13, −0.07)*	−0.06 (−0.09, −0.03)*	−0.07 (−0.10, −0.03)*	−0.10 (−0.13, −0.07)*	−0.12 (−0.16, −0.09)*
		2	−0.07 (−0.11, −0.04)*	−0.04 (−0.08, −0.01)*	−0.05 (−0.08, −0.01)*	−0.07 (−0.11, −0.04)*	−0.10 (−0.14, −0.07)*
		3	−0.08 (−0.11, −0.04)*	−0.06 (−0.10, −0.02)*	−0.06 (−0.10, −0.02)*	−0.07 (−0.11, −0.03)*	−0.09 (−0.13, −0.05)*
SAF, per SD	3254	1	−0.09 (−0.12, −0.05)*	−0.06 (−0.09, −0.02)*	−0.08 (−0.11, −0.04)*	−0.09 (−0.13, −0.06)*	−0.10 (−0.13, −0.06)*
		2	−0.05 (−0.09, −0.01)*	−0.03 (−0.07, 0.01)	−0.05 (−0.09, −0.01)*	−0.05 (−0.09, −0.01)*	−0.06 (−0.10, −0.02)*
		3	−0.05 (−0.08, −0.01)*	−0.03 (−0.07, 0.01)	−0.05 (−0.09, −0.01)*	−0.04 (−0.08, −0.001)*	−0.05 (−0.09, −0.01)*
Duration of diabetes, per SD	574	1	−0.09 (−0.17, −0.01)*	−0.09 (−0.17, −0.01)*	−0.09 (−0.17, −0.01)*	−0.10 (−0.18, −0.02)*	−0.06 (−0.14, 0.03)
		2	−0.08 (−0.16, −0.001)*	−0.09 (−0.17, −0.001)*	−0.09 (−0.17, −0.01)*	−0.09 (−0.17, −0.01)*	−0.05 (−0.13, 0.04)
		3	−0.09 (−0.17, −0.001)*	−0.10 (−0.18, −0.01)*	−0.09 (−0.18, −0.01)*	−0.09 (−0.18, −0.01)*	−0.04 (−0.13, 0.04)

Values are standardised regression coefficients (95% CI) representing the differences in corneal nerve fibre measures in SD for prediabetes or type 2 diabetes vs normal glucose metabolism or per SD greater for measures of glycaemia

For the glucose metabolism status, fasting plasma glucose, 2 h post-load glucose, HbA_1c_, SAF and duration of diabetes (type 2 diabetes only) study populations, 1 SD corresponds to 0.9 (unit-less) for the composite *Z*-score for corneal nerve fibre measures, 39.9 branches/mm^2^ for corneal nerve bifurcation density, 24.3 main and branches/mm^2^ for corneal nerve density, 4.4 mm/mm^2^ for corneal nerve length, 0.1 (unit-less) for corneal nerve fractal dimension, 1.4 mmol/l for fasting plasma glucose, 3.9 mmol/l for 2 h post-load glucose, 0.8% or 8.4 mmol/mol for HbA_1c_, 0.5 arbitrary units for SAF and 7.4 years for the duration of diabetes

Variables entered into models: model 1, adjusted only for corneal confocal microscopy visit interval; model 2, model 1 + age, sex and educational status (high, medium, low); model 3, model 2 + office systolic BP, total cholesterol to HDL-cholesterol ratio, use of antihypertensive and/or lipid-modifying medication (yes/no), waist circumference, smoking status (never, former, current) and alcohol consumption status (none, low, high)

Asterisks indicate values that are statistically significant (*p*<0.05)

### Measures of glycaemia and corneal nerve fibre measures

According to model 3, higher levels of measures of glycaemia (fasting plasma glucose, 2 h post-load glucose, HbA_1c_, SAF and duration of diabetes) were all significantly associated with a lower composite *Z*-score for corneal nerve fibre measures (per SD: −0.09 [−0.13, −0.05], −0.07 [−0.11, −0.03], −0.08 [−0.11, −0.04], −0.05 [−0.08, −0.01], −0.09 [−0.17, −0.001], respectively) (Table 2 and Fig. 2). In general, directionally similar associations were observed for individual corneal nerve fibre measures (Table 2).

### Interaction analyses

Sex modified the association of prediabetes vs normal glucose metabolism and composite *Z*-score for corneal nerve fibre measures (*p* value for the interaction term prediabetes × sex = 0.01). In women, but not in men, we found a significant association of prediabetes vs normal glucose metabolism with the composite *Z*-score for corneal nerve fibre measures (−0.18 [−0.33, −0.04] in women and 0.04 [−0.10, 0.19] in men). Stratified analyses for all associations are shown in ESM Table 4. However, the sex interaction stratified analyses did not consistently demonstrate significant sex differences in the associations between (pre)diabetes, measures of glycaemia and corneal nerve measures.

### Additional analyses

Quantitatively similar results were observed for a range of additional analyses (ESM Results and ESM Tables 3–13).

## Discussion

The present population-based observational study had the following main findings. First, after adjustment for a range of potential confounders, a more adverse glucose metabolism status and higher levels of glycaemic measures (fasting plasma glucose, 2 h post-load glucose, HbA_1c_, SAF and duration of diabetes) were all linearly associated with a lower composite *Z*-score for corneal nerve fibre measures. Second, associations were linear across glucose metabolism categories, implying that glycaemia-associated corneal nerve damage starts well before the onset of type 2 diabetes. Third, to put our findings into perspective, the *β* coefficients for prediabetes and type 2 diabetes correspond to 10 and 17 years of additional ageing, respectively (ESM Table 13).

To our knowledge, our study is the first large population-based study that investigated glycaemia-associated corneal nerve damage across glucose metabolism categories with adjustment for an extensive set of potential confounders. Our results are in line with observations from previous research [21, 31–40]. Novel associations that this study reports are the associations of 2 h post-load glucose and SAF with corneal nerve indices. In addition, the present study is, to our knowledge, the first to demonstrate that fasting plasma glucose, 2 h post-load glucose, SAF and duration of diabetes are all linearly associated with corneal nerve indices.

Our findings imply that the process of glycaemia-induced corneal neurodegeneration starts well before the onset of type 2 diabetes. Thus, these findings are in line with the ‘ticking clock hypothesis’ [5–9], which states that glycaemia-induced microvascular and neuronal deterioration is a continuous process that starts long before the onset of type 2 diabetes and gradually worsens during prediabetes and early and advanced type 2 diabetes [4]. Our results show a gradual decrease in the composite *Z*-score for corneal neurodegeneration from the reference group (with normal glucose metabolism) to prediabetes and towards type 2 diabetes, which is directionally comparable to previous findings on retinal nerve fibre layer thickness [5], brain structural abnormalities [6, 7], peripheral nerve function [8] and heart rate variability [9], which are all measures of neurodegeneration and/or neural dysfunction.

Additionally, our findings suggest that AGEs may be implicated in the pathobiology of corneal neurodegeneration. Indeed, we demonstrated that SAF, which reflects the accumulation of AGEs in the skin, was significantly associated with a lower composite *Z*-score for corneal nerve fibre measures even after adjustment for fasting plasma glucose, 2 h post-load glucose or HbA_1c_ (ESM Table 11).

We used a composite score (i.e. composite *Z*-score for corneal nerve fibre measures) to determine corneal neurodegeneration. The advantage of this approach is that the influence of the technical or biological variability is reduced [41] provided that the associations between the key exposure (here glucose) and all elements of the *Z*-score are directionally similar, which is the case here: when we investigated the associations between glucose metabolism status and measures of glycaemia with the corneal nerve fibre measures individually (corneal nerve bifurcation density, corneal nerve density, corneal nerve length and fractal dimension), we found directionally similar associations (Table 2).

Note that the corneal nerve measures in our study population differ from those in other studies [42], possibly because (1) we assessed the corneal nerve variables in larger images, with an area up to 1600×1600 µm, and therefore partially including the inferior whorl in some of the composite images, which is characterised by a different architecture, distribution and density of the corneal nerve fibres [43], (2) we used a different deep learning model (U-Net-based convolutional neural network) to fully automatically trace and analyse the indices of corneal nerves [44], and (3) the corneal nerve density and length were also defined differently from other studies (total number and length of corneal nerves, both main and branches, per mm^2^). However, these outcomes were assessed in all sub-groups of participants and therefore the differences do not detract from the consistency of our findings.

Although the association between prediabetes and a lower composite *Z*-score for corneal nerve measures, in model 3, was not significant when compared with normal glucose metabolism, this is probably due to insufficient statistical power. Therefore, we tested the linear trend with glucose metabolism status deterioration, using the statistically more powerful *p* value to estimate the linear trend, and observed that a more adverse glucose metabolism status was consistently associated with a linear decrease in the composite *Z*-score for corneal nerve fibre measures. These associations are consistent with our findings that showed linear associations of continuous glycaemia measures with corneal nerve fibres (Table 2).

Our findings may have implications for clinical practice. First, early glycaemic control, before the onset of type 2 diabetes, is likely to be important in prevention of early neurodegeneration. Second, because corneal neurodegeneration may be a biomarker for diabetic neuropathy [45], detection of early stages of neuropathy is relatively inexpensive and easy to perform.

Our study had several strengths. First, it is, to our knowledge, the first large population-based observational study with oversampling of individuals with type 2 diabetes that studied the associations between (pre)diabetes and measures of glycaemia with corneal neurodegeneration. Second, we adjusted for a large series of potential confounders. Finally, additional analyses generally yielded consistent findings.

We suggest that the composite *Z*-score for corneal nerve measures may be a potential predictor for neuropathy, independent of glucose metabolism status. Our results showed numerically similar correlation coefficients for individuals in all glucose metabolism strata, although the smaller sample size of individual groups led to lower statistical power than for the main study population. In addition, we performed an additional analysis observing the associations of prediabetes and type 2 diabetes vs normal glucose metabolism with the composite *Z*-score for corneal nerve fibre measures after exclusion of participants with sensory neuropathy. After adjustment (model 3), a more adverse glucose metabolism status was associated with a lower composite *Z*-score for corneal nerve fibre measures (*β* [95% CI]: type 2 diabetes vs normal glucose metabolism −0.16 [−0.27, −0.04], prediabetes vs normal glucose metabolism −0.08 [−0.19, 0.03]) (data not shown). These associations suggest that corneal neurodegeneration starts before the development of clinical sensory neuropathy.

Our study also had limitations. First, because of the observational cross-sectional nature of the study, any causal inferences should be made with caution. Nevertheless, reverse causation appears unlikely, and we adjusted for many potential confounders. Second, underestimation of the association of prediabetes vs normal glucose metabolism with composite *Z*-score for corneal nerve fibre measures may have occurred due to misclassification of individuals with prediabetes, as the classification was based on one OGTT only. Third, we may have underestimated associations due to range restriction for analyses involving 2 h post-load glucose, as individuals who used insulin or had a fasting plasma glucose concentration above 11.0 mmol/l did not undergo an OGTT. Fourth, even though we considered an extensive number of confounders, we cannot fully exclude bias due to unmeasured confounding, for example by environmental factors such as air pollution. Fifth, because corneal confocal microscopy measurements started from April 2013, individuals who participated in the Maastricht Study before the start of corneal confocal microscopy measurement (i.e. before April 2013; *n*=974; 28.1%) had a median visit interval of 5.2 years (IQR 4.9–5.8). However, to provide a more appropriate estimate of the true association, we adjusted for visit interval in model 1 for all associations. We also analysed potential interaction with visit interval. We added an interaction term (i.e. fasting plasma glucose × visit interval) [30] to model 3 (data not shown). The interval did not modify the association between fasting plasma glucose and composite *Z*-score for corneal nerve fibre measures (*p* value of the interaction term fasting plasma glucose × visit interval = 0.16). We also performed additional analyses in which we excluded participants with a catch-up visit to assess the association between fasting plasma glucose and *Z*-score for corneal nerve fibre measures. Similar results were observed (data not shown). Finally, this study comprised individuals of European descent aged between 40 and 75 years old with access to high-quality diabetes care. Therefore, further study is required to determine whether our results may be generalised to other populations.

### Conclusions

In summary, the present population-based study demonstrated that a more adverse glucose metabolism status and a higher level of glycaemic measures are continuously associated with lower composite *Z*-score for corneal nerve fibre measures. These associations were independent of demographics, cardiovascular or lifestyle risk factors. Hence, our results indicate that glycaemia-associated morphological changes in the corneal nerves may be an early process that starts well before the onset of type 2 diabetes. Whether early intervention to reduce hyperglycaemia can prevent corneal neurodegeneration requires further study.

### Supplementary Information

Below is the link to the electronic supplementary material.

Supplementary file1 (PDF 861 KB)

## Data Availability

Data from the Maastricht Study are available to any researcher who meets the criteria for access to confidential data. The corresponding author may be contacted to request access.

## Abbreviations

AGE: Advanced glycation end-product
SAF: Skin autofluorescence
