# Supplementary material for: (Pre)diabetes and a higher level of glycaemic measures are continuously associated with corneal neurodegeneration assessed by corneal confocal microscopy: the Maastricht Study
Source: Diabetologia. 2023 Aug 17;66(11):2030–41. doi: 10.1007/s00125-023-05986-5 (PMC10541833; doi:10.1007/s00125-023-05986-5)
Supplement: Supplementary file 1 — Supplementary file1 (PDF 861 KB) [file 125_2023_5986_MOESM1_ESM.pdf]

(Pre)diabetes and a higher level of glycaemic measures are continuously associated with corneal neurodegeneration assessed by corneal confocal microscopy: the Maastricht Study

Sara B.A. Mokhtar[1,2,3,4]; Frank C.T. van der Heide[1,2,3,4]; Karel A.M. Oyaert[1,2,3]; Carla J.H. van der Kallen[1,2]; Tos T.J.M. Berendschot[3,4]; Fabio Scarpa[5]; Alessia Colonna[5]; Bastiaan E. de Galan[1,2,6]; Marleen M.J. van Greevenbroek[1,2]; Pieter C. Dagnelie[1,2]; Casper G. Schalkwijk[1,2]; Rudy M.M.A. Nuijts[4]; Nicolaas C. Schaper[1,7,8]; Abraham A. Kroon[1,2,9]; Miranda T. Schram[1,2,9]; Carroll A.B. Webers[4]; Coen D.A. Stehouwer[1,2]

Sara B.A. Mokhtar and Frank C.T. van der Heide contributed equally to this study

[1]CARIM School for Cardiovascular Diseases, Maastricht University, the Netherlands
[2]Department of Internal Medicine, Maastricht University Medical Center, the Netherlands
[3]School of Mental Health and Neuroscience, Maastricht University, the Netherlands
[4]University Eye Clinic Maastricht, Maastricht University Medical Center, the Netherlands
[5]Department of Information Engineering, University of Padova, Italy
[6]Department of Internal Medicine, Radboud University Medical Center, Nijmegen, the Netherlands
[7]Department of Internal Medicine, Division of Endocrinology and Metabolic Disease, Maastricht University Medical Center, the Netherlands
[8]Care and Public Health Research Institute, Maastricht University, the Netherlands
[9]Heart and Vascular Center, Maastricht University Medical Center, the Netherlands

Corresponding author: Sara Mokhtar, s.mokhtar@maastrichtuniversity.nl

Supplemental Material

Content

ESM Methods

ESM Results

ESM Figures

ESM Tables

Abbreviations

AGE Advanced glycation end-product

SAF Skin autofluorescence

Content

- **ESM Table 1** Additional general study population characteristics according to tertiles of composite Z-score for corneal nerve fibre measures in the study population with complete data on glucose metabolism status
- **ESM Table 2** General study population characteristics of the included and excluded participants
- **ESM Table 3** Associations of glucose metabolism status and measures of glyacemia with corneal nerve fibre measures expressed as regression coefficients (β)
- **ESM Table 4** P-values for the interaction term of sex with determinants under the study and analyses stratified by sex
- **ESM Table 5** Associations of glucose metabolism status and measures of glycaemia with composite Z-score for corneal nerve fibre measures additionally adjusted for life style factors (dietary intake (except for alcohol) and physical activity, model 4A), for ocular variables (corneal diseases, uveitis, and use of contact lenses, model 4B), or for cardiovascular risk factors (kidney variables, history of cardiovascular disease, and low-grade inflammation biomarkers, model 4C)
- **ESM Table 6** Associations of glucose metabolism status and measures of glycaemia with composite Z-score for corneal nerve fibre measures after exclusion of individuals with a history of corneal diseases, uveitis, or who used contact lenses (model 5)
- **ESM Table 7** Associations of glucose metabolism status and measures of glycaemia with corneal nerve measures, where waist circumference was replaced with BMI (model 6A), or office systolic blood pressure was replaced with office diastolic blood pressure (model 6B), with systolic 24-hour ambulatory blood pressure (model 6C), or with diastolic 24-hour ambulatory blood pressure (model 6D), or educational level was replaced with occupational status (model 6E), or income level (model 6F)
- **ESM Table 8** Associations of glucose metabolism status and measures of glycaemia with corneal nerve fibre absolute tortuosity (model 7A), corneal nerve fibre weighted tortuosity (model 7B), and corneal nerve length divided by absolute tortuosity (model 7C)
- **ESM Table 9** Associations of glucose metabolism status and measures of glycaemia with composite Z-score for corneal nerve fibre measures additionally adjusted for: location of captured corneal nerve images (model 8A), the orientation of the corneal

nerve fibres (model 8B), the total surface area of the captured image (model 8C), or where we included images with quality criteria of 25% or more (model 8D)

- **ESM Table 10** Associations of glucose metabolism status with corneal nerve measures stratified by image location center, semi-center, or inferior whorl
- **ESM Table 11** Standardized regression coefficients of SAF with composite Z-score for corneal nerve fibre measures after additional adjustment for fasting plasma glucose, 2-hour post-load glucose, or $HbA1_c$
- **ESM Table 12** correlation of sensory neuropathy and vibration perception with composite Z-score for corneal nerve fibre measure
- **ESM Table 13** Associations of age with composite Z-score for corneal nerve fibre measure

ESM Methods

Assessment of corneal confocal microscopy measurements (outcome)

We chose the left eye as a previous studies has shown that corneal nerve alterations are symmetrical in controls and subjects with diabetes. Therefore for logistic reasons to enable us to undertake corneal nerve images in such a large cohort we selected the left eye. Moreover, We captured the nerve fibres of the left eye only while both eyes were anesthetized because the corneal reflex causes both eyes to blink as a response to one eye stimulation (contact between the cap and the cornea), in this case the direct contact of the cap with the cornea.

The captured images were performed in the region of interest (subbasal nerve plexus). When imaging corneal nerves using the corneal confocal microscopy, the captured images are typically two-dimensional representations of the corneal nerve structure. These images are taken from the anterior surface of the cornea and provide a cross-sectional view of the nerve fibres in the X-Y plane. However, it is important to note that corneal nerves do have a three-dimensional distribution, with some fibres extending deeper into other corneal layers.

Advantage of performing large scale corneal confocal microscopy image (1600 × 1600 μm)

A single image (400 × 400 μm) captures only a small portion of the cornea, which is insufficient to provide a representative assessment of the overall nerve health throughout the entire cornea. The aim of creating a montage is to analyse a larger corneal region and properly handle any overlaps that occur when stitching multiple images together. We have referenced a specific composite algorithm implemented in the HRT3 user interface (Heidelberg Engineering) that explains the process used for image assembly [1]. This algorithm ensures accurate alignment and combination of individual images. Additionally, we employed a U-Net-Based Convolutional Neural Network to fully automate the tracing and analysis of corneal nerve indices per mm^2 [2]. This approach allows for normalization of the measured indices based on the depicted area.

Grading of corneal confocal microscopy images

According to the selection criteria, images were considered to be usable (n= 3623) if 50% or more of the total captured area was of good quality. For example, a fully captured area consists of 16 sub-squares. If at least 8 sub-squares were of good quality, the image was included. For assessment, we scored the segmented images (number of good quality sub-images) based on

the contrast, the depth, the sharpness, the focus position, and the presence of pressure lines (ESM Figure 2). We also assessed the location of the captured images based on the orientation of the corneal nerves (i.e., in the center of the cornea, corneal nerves are thought to be vertical (n=368), peripherally, corneal nerves are thought to be more horizontal (n=2694), and the Inferior whorl, corneal nerves are thought to show a swirl pattern (n=405), ESM Figure 3). The percentage of agreement of quality image assessment between observers was on average 97.2%.

Assessment of advanced glycation end-products (AGEs)

Due to the use of different versions of calibration software, absolute SAF values assessed before the 4th September 2012 were 0.5 arbitrary units higher than the SAF values assessed after this date. To realign absolute SAF values, we recalculated the SAF values assessed before the 4th of September 2012 by subtracting 0.5 arbitrary units from the SAF values assessed before the 4th September 2012.

Assessment of retinopathy

All signs of retinopathy were graded from fundus photos by an ophthalmologist according the Early Treatment Diabetic Retinopathy Study Research Group (ETDRS) criteria [3]. Presence of retinopathy was defined according to the American Academy of ophthalmology preferred practice guidelines [4].

Assessment of Dutch Healthy Diet Score

The Dutch Healthy Diet score 2015 (DHD-score) consists of fifteen components representing the fifteen food-based Dutch dietary guidelines updated in 2015 [5]. For each food component, the score ranged between 0 and 10 points. For a healthy food component (adequacy component, i.e., vegetables, fruits, whole grain products, legumes, nuts, fish, tea), intake equal to or higher than a cut-off value, specified according to the dietary guidelines, the maximum score was given, whilst for consumption below that cut-off value the score was calculated by means of linear interpolation between threshold value (often 0, score = 0) and cut-off value (score = 10). A detailed description of the operationalization has been described previously [5]. We recalculated the Dutch Healthy Diet score so that the “diet score” reflects dietary intake without alcohol consumption.

Additional analyses

First, we repeated the analyses with additional adjustment for lifestyle factors (dietary intake and physical activity) and for ocular diseases (history of corneal diseases, uveitis), and use of contact lenses (ESM Table 5). Adjustments for these potential confounders were not included in the main analyses because data were missing for a relatively large number of participants. Second, we additionally adjusted for kidney variables (eGFR and urinary albumin excretion), a history of cardiovascular disease, and plasma biomarkers of low-grade inflammation (ESM Table 5). We adjusted for these covariates in a separate model because they may be confounders but may also (in part) be mediators or descendants of the outcome. Third, we performed additional analyses where we excluded participants with eye diseases (history of corneal diseases, uveitis) or who used contact lenses (ESM Table 6). Fourth, we replaced waist circumference with BMI, office systolic blood pressure with office diastolic blood pressure or systolic or diastolic 24-hour ambulatory blood pressure, and education level with income level or occupational status (ESM Table 7). Fifth, we studied the associations of potential determinants with corneal nerve fibre tortuosity (absolute tortuosity, and weighted tortuosity [absolute tortuosity divided by length]) and with corneal nerve length divided by absolute tortuosity, since corneal nerve length increases by increasing tortuosity [6] (ESM Table 8). Sixth, we performed additional analyses in which we adjusted for the orientation of the corneal nerve fibre (entered as continuous variable) or for the location of measured corneal nerve fibre images (entered as dummy variables of center, semi-center, and inferior whorl) (ESM Table 9, Model 8A, and 8B). We analysed stratified by location associations to study potential differences according to cornea scan location. We did this because the features of corneal nerves are different according to the location of the acquired image (e.g., the corneal nerve density is higher in the inferior whorl compared to the center or the semi-center). Seventh, we studied the associations between determinants under the study with composite Z-score for corneal nerve fibre measures additionally adjusted for the total area of the acquired image (surface area) to study a possible role of inhomogeneous distribution of nerve fibres over the cornea, as the size of the surface of the corneal nerve plexus that is measured may affect the number of nerve fibres that is observed (ESM Table 9, Model 8C). Eighth, because we only included images that had at least 50% of good quality, which resulted in a relatively large number of exclusions (n=532), we performed additional analyses where we included images with quality criteria of 25% or more (ESM Table 9, Model 8D). Ninth, we studied the associations between glucose metabolism status and all individual corneal nerve measures, including corneal nerve weighted tortuosity, stratified by image location (center, semi-center, and inferior whorl, more details about how we assessed the location of the corneal nerve fibres

are provided in the Supplemental Material) (ESM Table 10). Tenth, and only for SAF, we additionally adjusted the association between SAF and composite Z-score for corneal nerve fibre measures for fasting plasma glucose, 2-hour post-load glucose, or $HbA1_c$. We additionally adjusted for these measures to investigate whether the association between SAF and composite Z-score for corneal nerve fibre measures was independent of short to middle long-term exposure to hyperglycaemia (ESM Table 11). Eleventh, we assessed the point biserial correlation between sensory neuropathy and the composite Z-score for corneal nerve fibre measures. In addition, we analysed the partial correlation, after adjustment for age, between vibration perception (continuous variable) and composite Z-score for corneal nerve measures. We also repeated the same analysis in different glucose metabolism strata (ESM Table 12). Last, we studied the association between age and composite Z-score for corneal nerve fibre measures (ESM Table 13) to allow comparison of associations with prediabetes or type-2 diabetes and age.

Supplemental Results

Additional analyses

Quantitatively similar results were observed in a range of sensitivity analyses. First, associations were generally comparable to the main results after additional adjustment for lifestyle factors (dietary intake and physical activity), for ocular diseases (history of corneal diseases and uveitis), use of contact lenses, or for kidney variables (eGFR and urinary albumin excretion), a history of cardiovascular disease, and plasma biomarkers of low-grade inflammation (ESM Table 5). Second, associations were similar after excluding participants with eye diseases (history of corneal diseases and uveitis) or who used contact lenses (ESM Table 6). Third, associations remained similar after replacement of waist circumference with BMI, office systolic blood pressure with office diastolic blood pressure or systolic or diastolic 24-hour ambulatory blood pressure, and education level with income level or occupational status (ESM Table 7). Fourth, associations between the potential determinants and corneal nerve fibre tortuosity (absolute tortuosity and weighted tortuosity) were inconsistent compared with other corneal nerve fibre outcomes (ESM Table 8), while associations between the potential determinants and corneal nerve length divided by absolute tortuosity showed comparable results to corneal nerve length (ESM Table 8). Fifth, associations were not altered after additional adjustment for orientation of the corneal nerve fibre or for the location of measured corneal nerve fibre images (ESM Table 9, Model 8A, and 8B), for the total area of the acquired image (surface area) (ESM Table 9, Model 8C), or after including images with quality criteria of 25% or more (n=3996) (ESM Table 9, Model 8D). Sixth, associations of glucose metabolism status with composite Z-score for corneal nerve fibre measures, or with different corneal nerve fibre measures stratified by image location (center, semi-center, and inferior whorl), all showed stronger associations for images that were captured centrally (ESM Table 10). Seventh, the association between SAF was not altered after additional adjustment for FPG, 2-hour post-load glucose, or $HbA1_c$ (ESM Table 11). Eighth, our results indicate a significant negative correlation between sensory neuropathy and vibration perception with the composite Z-score for corneal nerve measures (r= -0.05, P=0.002, (95%CI) (-0.09, -0.01), r= -0.06, P value <0.001, (95%CI) (-0.09, -0.02), respectively). In general, directionally similar correlations were found for different glucose metabolism strata (ESM Table 12). Last, in model 3, we compared stβ of prediabetes and type-2 diabetes versus normal glucose metabolism with stβ for age to calculate the impact of having prediabetes and type-2 diabetes in additional aging

years. ESM Table 12 showed that having type-2 diabetes corresponds approximately with 17 years of aging.

References

[1] Allgeier S, Zhivov A, Eberle F, et al. (2011) Image reconstruction of the subbasal nerve plexus with in vivo confocal microscopy. Invest Ophthalmol Vis Sci 52(9): 5022-5028. 10.1167/iovs.10-6065

[2] Scarpa F, Colonna A, Ruggeri A (2020) Multiple-image deep learning analysis for neuropathy detection in corneal nerve images. Cornea 39(3): 342-347

[3] (1991) Grading diabetic retinopathy from stereoscopic color fundus photographs--an extension of the modified Airlie House classification. ETDRS report number 10. Early Treatment Diabetic Retinopathy Study Research Group. Ophthalmology 98(5 Suppl): 786-806

[4] Committee AAoOPPPRV (2019) Diabetic Retinopathy PPP 2019. Available from https://www.aao.org/preferred-practice-pattern/diabetic-retinopathy-ppp. Accessed 22nd of May 2020

[5] Looman M, Feskens EJ, de Rijk M, et al. (2017) Development and evaluation of the Dutch Healthy Diet index 2015. Public Health Nutr 20(13): 2289-2299. 10.1017/s136898001700091x

[6] Edwards K, Pritchard N, Vagenas D, Russell A, Malik RA, Efron N (2014) Standardizing corneal nerve fibre length for nerve tortuosity increases its association with measures of diabetic neuropathy. Diabetic Medicine 31(10): 1205-1209. https://doi.org/10.1111/dme.12466

ESM Figures

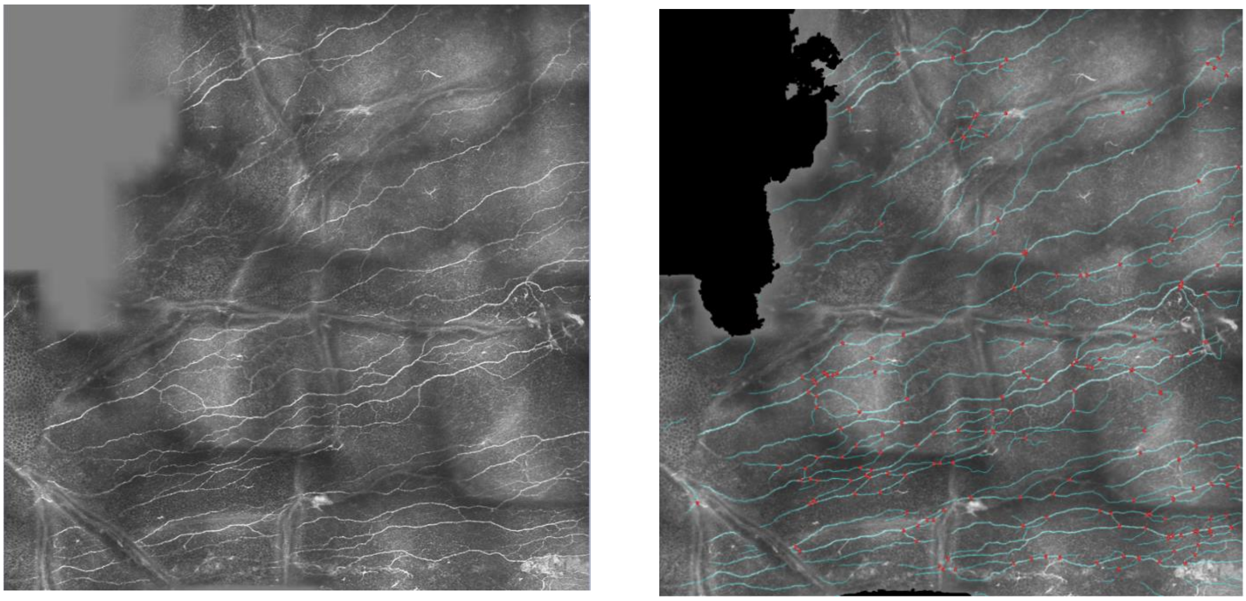

(a) **(b)**

ESM Figure 1 (a) Original CCM image (b) Automatically detected nerves

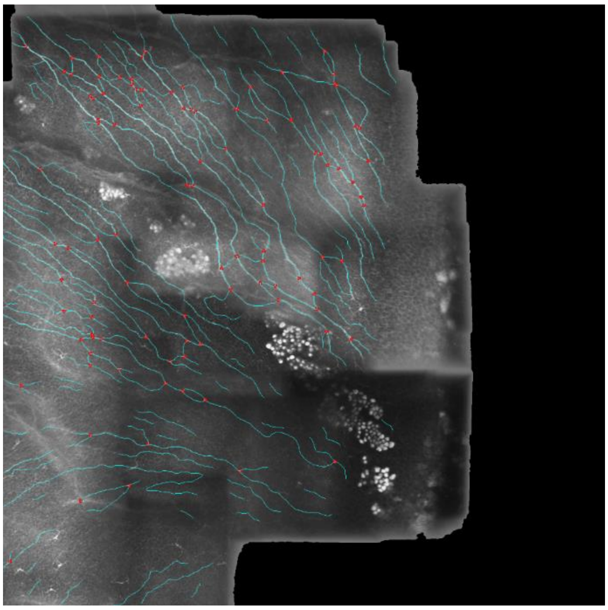

(a)

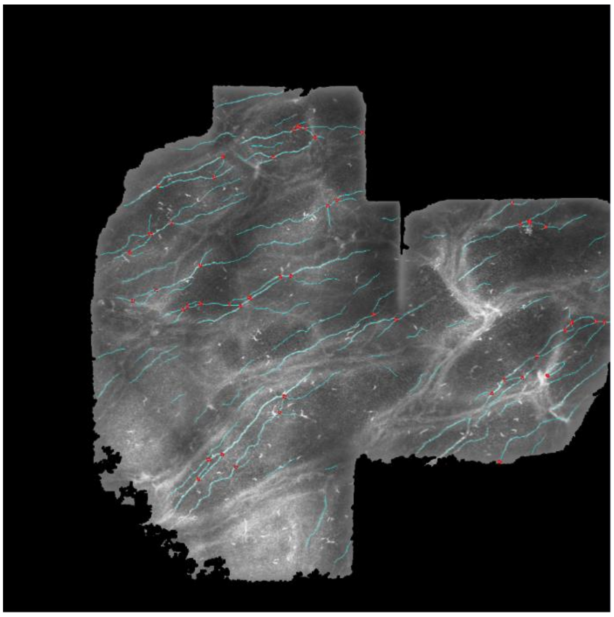

(b)

ESM Figure 2 Example of (a) Included image (b) Excluded image

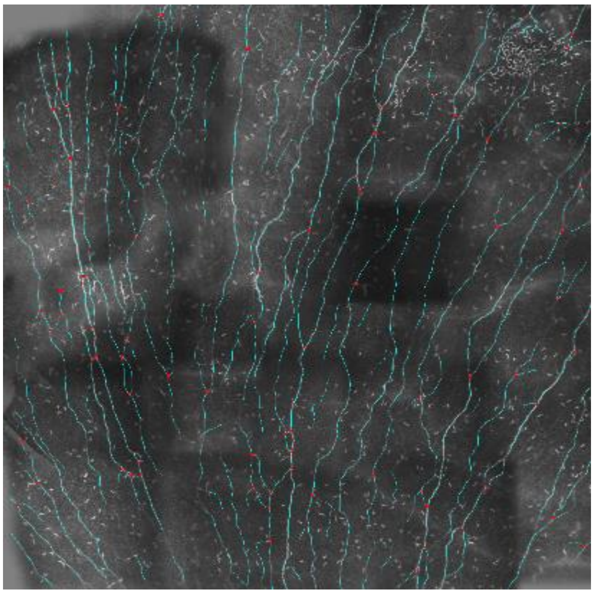

(a)

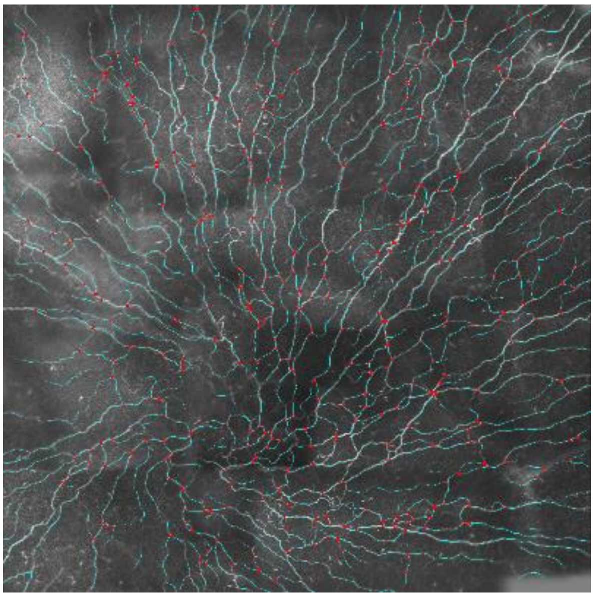

(b)

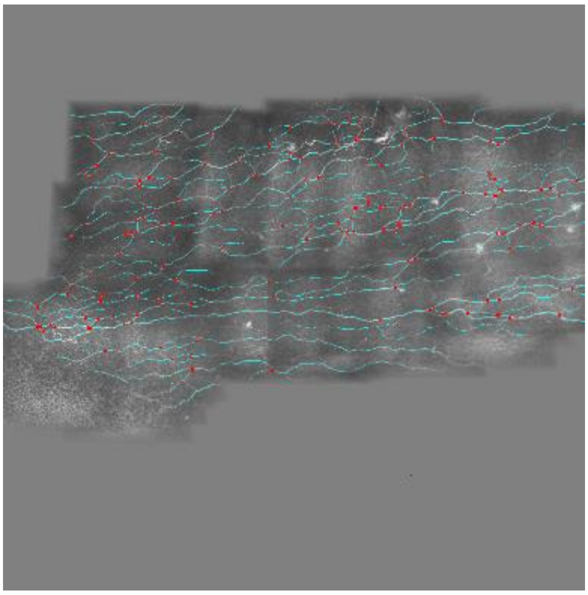

(c)

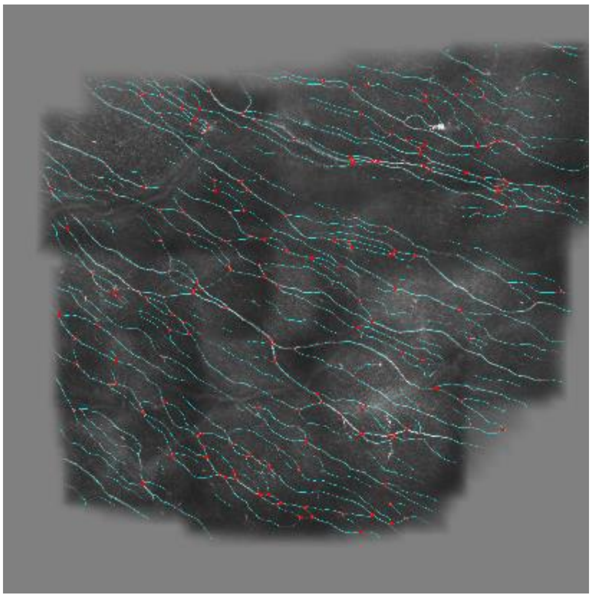

(d)

ESM Figure 3 (a) Center of the cornea (b) Inferior nasal (c and d) Peripheral

ESM Tables

ESM Table 1 Additional general study population characteristics according to tertiles of composite Z-score for corneal nerve fibre measures in the study population with complete data on glucose metabolism status

	Composite Z-score for corneal nerve fibre measures				
Characteristic	Total study population (n = 3471)	Tertile 1 (high) (n =1157)	Tertile 2 (middle) (n =1157)	Tertile 3 (low) (n =1157)	Number of participants with missing data
Body-mass index (kg/m^2)	26.7 ± 4.3	26.4 ± 4.0	26.9 ± 4.4	26.9 ± 4.4	0
Income level (euro)	1875 (503-2386)	1938 (1503-2386)	1856 (1503-2449)	1875 (1503-2386)	765
Occupational status					2733
High	243 (32.9)	58 (30.4)	77 (32.5)	108 (34.8)	
Intermediate	234 (31.7)	74 (38.7)	64 (27.0)	96 (31.0)	
Low	261 (35.4)	59 (30.9)	96 (40.5)	106 (34.2)	
Systolic ambulatory 24-hour blood pressure (mmHg)	118.6 ± 11.5	117.3 ± 10.9	119.3 ± 11.7	119.3 ± 11.8	311
Diastolic ambulatory 24-hour blood pressure (mmHg)	73.0 ± 7.3	72.7 ± 7.2	72.3 ± 7.3	73.0 ± 7.4	311
Dutch Healthy diet score (points)	77.1 ± 14.5	77.3 ± 14.2	77.4 ± 14.6	76.5 ± 14.8	239
Physical activity (hours/day)	2.0 ± 0.7	2.0 ± 0.7	2.0 ± 0.7	2.0 ± 0.7	370
Ocular disorder, yes vs. no					
Corneal diseases	42 (1.4)	11 (1.0)	10 (1.0)	21 (2.1)	384
Uveitis	86 (2.7)	34 (3.2)	22 (2.1)	30 (2.8)	389
Retinopathy	49 (1.4)	12 (1.1)	15 (1.3)	22 (2.0)	82
Use of contact lenses	292 (10.4)	102 (10.5)	109 (11.4)	81 (9.0)	654
History of cardiovascular diseases, yes vs. no	539 (15.6)	189 (16.4)	167 (14.5)	183 (15.9)	12
Kidney variables					
Estimated glomerular filtration rate, ml/min/1.73m^2	87.8 ± 14.3	89.2 ± 14.0	88.7 ± 13.2	86.3 ± 15.1	2606
Urinary albumin excretion (mg/24 hours)	5.2 (3.4-9.5)	4.5 (3.2-8.8)	5.3 (3.4-9.3)	5.6 (3.4-10.8)	13

Albuminuria (mean albuminuria more than 30mg/24 hours)	220 (6.4)	55 (4.8)	80 (7.0)	85 (7.4)	13
Biomarkers of low-grade inflammation					2463
C-reactive protein, µg/ml	1.2 (0.6-2.6)	1.0 (0.6-2.1)	1.2 (0.6-2.7)	1.3 (0.6-2.7)	
Serum amyloid A, µg/ml	3.4 (2.0-5.4)	3.2 (2.0-4.7)	3.5 (2.1-6.1)	3.4 (2.0-5.5)	
Interleukin-6, pg/ml	0.6 (0.4-0.9)	0.5 (0.4-0.8)	0.6 (0.4-0.9)	0.6 (0.4-0.9)	
Interleukin-8, pg/ml	4.2 (3.3-5.4)	4.0 (3.2-5.2)	4.0 (3.4-5.2)	4.4 (3.5-5.5)	
Tumor necrosis factor α, pg/ml	2.2 (1.9-2.5)	2.1 (1.8-2.5)	2.1 (1.8-2.5)	2.2 (1.9-2.6)	
Corneal nerve fibre absolute tortuosity	3.1 ± 0.4	3.2 ± 0.4	3.1 ± 0.5	2.9 ± 0.6	0
Corneal nerve fibre weighted tortuosity	7.1 ± 1.8	7.5 ± 1.5	7.2 ± 1.8	6.5 ± 1.9	0

Data are presented as mean ± standard deviation, median (interquartile range) or number (%)

ESM Table 2 General study population characteristics of the included and excluded participants

**Characteristic**	Included study population (n = 3471)	Missing data in/excluded	Excluded study population (n =4534)
Age (years)	59.4 ± 8.7	0/0	60.11 ± 8.7
Men	1681 (48.4)	0/0	2340 (51.6)
Educational level		0/115	
High	1332 (38.4)		1656 (36.5)
Medium	956 (27.5)		1210 (26.7)
Low	1183 (34.1)		1553 (34.3)
Glucose metabolism status		0/0	
Normal glucose metabolism	2225 (64.1)		2620 (57.8)
Prediabetes	509 (14.7)		681 (15)
Type-2 diabetes	729 (21.0)		1,191 (26.3)
Other type of diabetes	8 (0.2)		42 (0.9)
Measures of glycaemia			
Fasting plasma glucose (mmol/L)	5.7 ± 1.4	1/0	6.0 ± 1.8
2-hour post-load glucose (mmol/L)	6.0 (4.9-8.3)	143/344	6.2 (5.0-8.9)
$HbA1_c$ (mmol/mol)	38.6 ± 8.4	3/11	40.5 ± 10.3
$HbA1_c$ (%)	5.7 ± 0.8	3/11	5.9 ± 0.9
SAF (arbitrary units)	2.1 ± 0.5	217/402	2.2 ± 0.5
Duration of diabetes	3.0 (0.0-8.3)	0/411	4.0 (1.0-11.0)
Waist circumference, (cm)	94.3 ± 13.2	0/5	96.0 ± 14.0
Total-to-HDL cholesterol ratio	3.4 (2.8-4.2)	0/6	3.4 (2.8-4.3)
Use of lipid-modifying medication, yes vs. no	1009 (29.1)	0/6	1543 (34.0)
Office systolic blood pressure (mmHg)	132.8 ± 17.6	0/3	134.3 ± 18.2
Office diastolic blood pressure (mmHg)	75.5 ± 9.9	1/3	75.5 ± 9.8
Use of antihypertensive medication, yes vs. no	1220 (35.1)	0/6	1803 (39.8)
Smoking status		0/64	
Never	1341 (38.6)		1618 (35.7)

Former	1711 (49.3)		2208 (48.7)
Current	419 (12.1)		644 (14.2)
Alcohol consumption		0/65	
None	632 (18.2)		842 (18.6)
Low	2077 (59.8)		2551 (56.3)
High	762 (22.0)		1076 (23.7)
Number of participants that had corneal confocal microscopy scans at catch-up visit	974 (28.1)	0/0	670 (14.8)
Lag-time of corneal confocal microscopy scans (not done at baseline) (years)	5.2 (4.9-5.8)	0/0	4.4 (3.8-5.4)
Corneal nerve measures		0/46[a]	
Corneal nerve bifurcation density	73.9 ± 39.9		80.9 ± 64.5
Corneal nerve density	79.6 ± 24.3		83.9 ± 35.5
Corneal nerve length	14.9 ± 4.4		15.0 ± 5.0
Corneal nerve fractal dimension	1.3 ± 0.1		1.4 ± 0.1

Data are presented as mean ± standard deviation, median (interquartile range), or number (%)

[a] Number of participants with missing data represents the number of participants that had corneal nerve images of sufficient quality and were excluded due to missing confounders

ESM Table 3 Associations of glucose metabolism status and measures of glycaemia with corneal nerve fibre measures expressed as regression coefficients (β)

Determinant	Number of participants	Model	Corneal nerve bifurcation density	Corneal nerve density	Corneal nerve length	Corneal nerve fractal dimension × 100[1]
**Glucose metabolism status**	3471					
Prediabetes versus normal glucose metabolism		1	-3.56 (-7.37, 0.25)	-2.96 (-5.29, -0.64)*	-0.59 (-1.00, -0.17)*	-1.09 (-1.10, -0.21)*
		2	-2.20 (-6.07, 1.66)	-1.95 (-4.31, 0.40)	-0.32 (-0.74, 0.10)	-0.66 (-1.55, 0.23)
		3	-2.68 (-6.70, 1.34)	-2.28 (-4.73, 0.17)	-0.30 (-0.73, 0.14)	-0.42 (-1.35, 0.50)
Type-2 diabetes versus normal glucose metabolism		1	-5.21 (-8.54, -1.88)*	-3.96 (-6.00, -1.93)*	-0.95 (-1.31, -0.58)*	-2.55 (-3.32, -1.78)*
		2	-3.01 (-6.50, 0.47)	-2.39 (-4.52, -0.27)*	-0.58 (-0.96, -0.20)*	-1.95 (-2.75, -1.14)*
		3	-2.25 (-8,38, -0.11)*	-3.27 (-5.79, -0.75)*	-0.55 (-0.99, -010)*	-1.52 (-2.47, -0.57)*
**Measure of glycaemia**						
Fasting plasma glucose	3470	1	-2.35 (-3.30, -1.39)*	-1.53 (-2.11, -0.94)*	-0.34 (-0.45, -0.24)*	-0.82 (-1.04, -0.60)*
		2	-1.74 (-2.74, -0.75)*	-1.08 (-1.69, -0.48)*	-0.24 (-0.35, -0.14)*	-0.65 (-0.88, -0.42)*
		3	-2.21 (-3.34, -1.07)*	-1.35 (-2.04, -0.66)*	-0.25 (-0.37, -0.13)*	-0.56 (-0.82, -0.30)*
2-hour post-load glucose	3328	1	-0.61 (-0.96, -0.27)*	-0.43 (-0.65, -0.22)*	-0.10 (-0.14, -0.07)*	-0.25 (-0.33, -0.17)*
		2	-0.44 (-0.80, -0.08)*	-0.31 (-0.53, -0.08)*	-0.07 (-0.11, -0.03)*	-0.20 (-0.28, -0.12)*
		3	-0.62 (-1.03, -0.20)*	-0.42 (-0.67, -0.16)*	-0.07 (-0.12, -0.03)*	-0.17 (-0.26, -0.07)*
$HbA1_c$	3468	1	-3.14 (-4.85, -1.42)*	-2.13 (-3.18, -1.08)*	-0.57 (-0.76, -0.38)*	-1.48 (-1.88, -1.09)*
		2	-2.24 (-4.02, -0.11)*	-1.46 (-2.54, -0.37)*	-0.41 (-0.60, -0.21)*	-1.21 (-1.62, -0.80)*
		3	-2.99 (-5.02, -0.95)*	-1.84 (-3.08, -0.60)*	-0.41 (-0.63, -0.19)*	-1.06 (-1.53, -0.59)*
SAF	3254	1	-4.79 (-7.76, -1.82)*	-4.12 (-5.93, -2.31)*	-0.89 (-1.22, -0.57)*	-1-95 (-2.65, -1.26)*
		2	-2.58 (-5.81, 0.65)	-2.56 (-4.52, -0.59)*	-0.47 (-0.83, -0.12)*	-1.23 (-1.98, -048)*
		3	-2.64 (-5.97, 0.68)	-2.48 (-4.51, -0.46)*	-0.39 (-0.76, -0.03)*	-0.97 (-1.74, -0.20)*
Duration of diabetes	574	1	-0.48 (-0.94, -0.04)*	-0.32 (-0.60, -0.04)*	-0.06 (-0.11, -0.02)*	-0.76 (-0.19, 0.04)
		2	-0.47 (-0.93, -0.02)*	-0.29 (-0.57, -0.02)*	-0.06 (-0.11, -0.01)*	-0.06 (-0.18, 0.05)
		3	-0.53 (-1.02, -0.06)*	-0.32 (-0.61, -0.03)*	-0.06 (-0.11, -0.001)*	-0.06 (-0.18, 0.06)

Values are presented as regression coefficients (95% CI) representing the increase in determinant per increase of corneal nerve fibre measure (outcome)

Variables entered into the models: model 1: adjusted only for ‘corneal confocal microscopy visit interval’, model 2: model 1 + age, sex, and educational status (high, medium, low), model 3: model 2 + office systolic blood pressure, total cholesterol-to-HDL cholesterol ratio, use of antihypertensive and/ or lipid-modifying medication (yes/no), waist circumference, smoking status (never, former, current), and alcohol consumption status (none, low, high)

[1] The coefficients have been multiplied by 100 for ease of interpretation. The original associations between glucose metabolism status, measures of glycaemia, and corneal nerve fractal dimension yielded coefficients that were very close to zero and contained numerous decimal places

Asterisks indicate values that are statistically significant ($p<0.05$)

ESM Table 4 P-values for the interaction term of sex with determinants under the study and analyses stratified by sex

			Men		Women	
Determinant	Number of participants	P-value (Sex*determinant)	Number of participants	Composite Z-Score for corneal nerve fibre measures	Number of participants	Composite Z-Score for corneal nerve fibre measures
**Glucose metabolism status**	3463[a]		1677[a]		1786[a]	
Prediabetes versus normal glucose metabolism	509 / 2,225[b]	0.01*	264 / 934[b]	0.04 (-0.10, 0.19)	245 / 1291[b]	-0.18 (-0.33, -0.04)*
Type-2 diabetes versus normal glucose metabolism	729 / 2,225[c]	0.053	479 / 934[c]	-0.12 (-0.26, 0.01)	250 / 1291[c]	-0.16 (-0.32, 0.00)
**Measure of glycaemia**						
Fasting plasma glucose	3470	0.27	1681	-0.07 (-0.12, -0.02)*	1789	-0.10 (-0.15, -0.04)*
2-hour post-load glucose	3328	0.44	1591	-0.06 (-0.12, -0.01)*	1737	-0.08 (-0.13, -0.02)*
$HbA1_c$	3468	0.51	1681	-0.07 (-0.12, -0.02)*	1787	-0.08 (-0.13, -0.02)*
SAF	3254	0.050	1564	-0.09 (-0.15, -0.04)*	1690	-0.01 (-0.06, 0.04)
Duration of diabetes	574	0.17	375	-0.13 (-0.24, -0.03)*	199	-0.001 (-0.13, 0.14)

p-values represent the *p*-values for the interaction terms of sex with determinant under the study (e.g., sex × $HbA1_c$) in the associations of glucose metabolism status and measures of glycaemia with corneal nerve fibre measures. Variables in the model in addition to determinants and interaction term(s) with sex are: corneal confocal microscopy visit interval, age, sex, educational status, office systolic blood pressure, total cholesterol-to-HDL cholesterol ratio, use of antihypertensive and/ or lipid-modifying medication, waist circumference, smoking status, and alcohol consumption status. In addition, we added an interaction term for all confounders (e.g., age × sex, systolic blood pressure × sex)

Values are standardised regression coefficients (95% CI) representing the differences in corneal nerve fibre measures in SD for prediabetes or type 2 diabetes vs normal glucose metabolism or per SD greater for measures of glycaemia

For the glucose metabolism status, fasting plasma glucose, 2-hour post-load glucose, $HbA1_c$, SAF, and duration of diabetes (only for type-2 diabetes) study populations, 1 SD for men corresponds with 0.9 (unit-less) for composite Z-score for corneal nerve measures, 1.5 mmol/L for fasting plasma glucose, 4.3 mmol/L for 2-hour post-load glucose, 0.9 % or 9.3 mmol/mol for $HbA1_c$, 0.5 arbitrary units for SAF, and 7.5 years for the duration of diabetes. 1 SD for women corresponds with 0.9 (unit-less) for composite Z-score for corneal nerve measures, 1.2 mmol/L for fasting plasma glucose, 3.5 mmol/L for 2-hour post-load glucose, 0.7% or 7.4 mmol/mol for $HbA1_c$, 0.4 arbitrary units for SAF, and 7.1 years for the duration of diabetes

Variables entered into model 3: adjusted for 'corneal confocal microscopy visit interval', age, sex, educational status (high, medium, low), office systolic blood pressure, total cholesterol-to-HDL cholesterol ratio, use of antihypertensive and/ or lipid-modifying medication (yes/no), waist circumference, smoking status (never, former, current), and alcohol consumption status (none, low, high)

[a] Number of participants with normal glucose metabolism, prediabetes, and type-2 diabetes. Other types of diabetes (n=8) were excluded because of the small number of participants

[b] Number of participants with prediabetes versus number of participants with normal glucose metabolism

[c] Number of participants with type-2 diabetes versus number of participants with normal glucose metabolism

ESM Table 5 Associations of glucose metabolism status and measures of glycaemia with the composite Z-score for corneal nerve fibre measures additionally adjusted for life style factors (dietary intake (except for alcohol) and physical activity, model 4A), for ocular variables (corneal diseases, uveitis, and use of contact lenses, model 4B), or for cardiovascular risk factors (kidney variables, history of cardiovascular disease, and low-grade inflammation biomarkers, model 4C)

Determinant	Number of participants	Composite Z-score for corneal nerve fibre measures	Number of participants	Composite Z-score for corneal nerve fibre measures	Number of participants	Composite Z-score for corneal nerve fibre measures
		stβ (95% CI)		stβ (95% CI)		stβ (95% CI)
		4A		4B		4C
**Glucose metabolism status**	2888		2371		848	
Prediabetes versus normal glucose metabolism	423/ 1837[a]	-0.12 (-0.23, -0.01)*	349/ 1571[a]	-0.03 (-0.15, 0.09)	123/ 495[a]	-0.18 (-0.38, 0.03)
Type-2 diabetes versus normal glucose metabolism	628/ 1837[b]	-0.15 (-0.26, -0.04)*	451/ 1571[b]	-0.17 (-0.30, -0.04)*	230/ 495[b]	-0.27 (-0.48, -0.06)*
**Measure of glycaemia**						
Fasting plasma glucose, per SD	2894	-0.09 (-0.13, -0.05)*	2374	-0.12 (-0.17, -0.07)*	850	-0.09 (-0.17, -0.02)*
2-hour post-load glucose, per SD	2769	-0.08 (-0.12, -0.03)*	2300	-0.07 (-0.12, -0.02)*	806	-0.12 (-0.20, -0.03)*
$HbA1_c$, per SD	2892	-0.08 (-0.12, -0.03)*	2371	-0.10 (-0.15, -0.05)*	848	-0.07 (-0.15, 0.02)
SAF, per SD	2714	-0.05 (-0.09, -0.001)*	2217	-0.05 (-0.10, -0.001)*	827	-0.03 (-0.11, 0.05)
Duration of diabetes, per SD	495	-0.12 (-0.21, -0.03)*	336	-0.09 (-0.21, 0.02)	187	-0.04 (-0.21, 0.12)

Standardized regression coefficients (stβ) represent the differences in composite Z-score for corneal nerve fibre measures in SD for prediabetes or type-2 diabetes versus normal glucose metabolism or per SD greater measure of glycaemia. In the glucose metabolism status, fasting plasma glucose, 2-hour post-load glucose, $HbA1_c$, SAF, and duration of diabetes (only for type-2 diabetes) study populations, 1 SD corresponds with 0.9 (unit-less) for composite Z-score for corneal nerve fibre measures, 1.4 mmol/L for fasting

plasma glucose, 3.9 mmol/L for 2-hour post-load glucose, 0.8 % or 8.4 mmol/mol for $HbA1_c$, 0.5 arbitrary units for SAF, and 7.4 years for the duration of diabetes. In Models 4A, 4B, and 4C values per SD were numerically comparable

Asterisks indicate values that are statistically significant ($p<0.05$)

Variables entered into the models: CCM visit interval, age, sex, and educational status (high, medium, low), office systolic blood pressure, total cholesterol-to-HDL cholesterol ratio, use of antihypertensive or lipid-modifying medication (yes/no), waist circumference, smoking status (never, former, current), and alcohol consumption status (none, low, high)

[a] Number of participants with prediabetes versus number of participants with normal glucose metabolism

[b] Number of participants with type-2 diabetes versus number of participants with normal glucose metabolism

ESM Table 6 Associations of glucose metabolism status and measures of glycaemia with the composite Z-score for corneal nerve fibre measures after exclusion of individuals with a history of corneal diseases, uveitis, or who used contact lenses (model 5)

Determinant	Number of participants	Composite Z-score for corneal nerve fibre measures<br>stβ (95% CI)<br>Model 5
**Glucose metabolism status**	2093	
Prediabetes versus normal glucose metabolism	315 / 1329[a]	-0.03 (-0.16, 0.10)
Type-2 diabetes versus normal glucose metabolism	449 / 1329[b]	-0.14 (-0.27, -0.01)*
**Measure of glycaemia**		
Fasting plasma glucose, per SD	2096	-0.11 (-0.16, -0.05)*
2-hour post-load glucose, per SD	2021	-0.07 (-0.12, -0.02)*
$HbA1_c$, per SD	2094	-0.10 (-0.15, -0.04)*
SAF, per SD	1955	-0.05 (-0.11, -0.001)*
Duration of diabetes, per SD	341	-0.12 (-0.24, 0.01)

Standardized regression coefficients (stβ) represent the differences in composite Z-score for corneal nerve fibre measures in SD, per SD for greater index of glucose variability or for prediabetes or type-2 diabetes versus normal glucose metabolism or per SD greater measure of glycaemia. In the glucose metabolism status, fasting plasma glucose, 2-hour post-load glucose, $HbA1_c$, SAF, and duration of diabetes (only for type-2 diabetes) study populations, 1 SD corresponds with 0.9 (unit-less) for composite Z-score for corneal nerve fibre measures, 1.4 mmol/L for fasting plasma glucose, 3.9 mmol/L for 2-hour post-load glucose, 0.8% or 8.4 mmol/mol for $HbA1_c$, 0.5 arbitrary units for SAF, and 7.3 years for the duration of diabetes

Asterisks indicate values that are statistically significant ($p<0.05$)

Variables entered into the models: CCM visit interval, age, sex, and educational status (high, medium, low), office systolic blood pressure, total cholesterol-to-HDL cholesterol ratio, use of antihypertensive or lipid-modifying medication (yes/no), waist circumference, smoking status (never, former, current), and alcohol consumption status (none, low, high)

[a] Number of participants with prediabetes versus number of participants with normal glucose metabolism

[b] Number of participants with type-2 diabetes versus number of participants with normal glucose metabolism

ESM Table 7 Associations of glucose metabolism status and measures of glycaemia with corneal nerve measures, where waist circumference was replaced with BMI (model 6A), or office systolic blood pressure was replaced with office diastolic blood pressure (model 6B), with systolic 24-hour ambulatory blood pressure (model 6C), or with diastolic 24-hour ambulatory blood pressure (model 6D), or educational level was replaced with occupational status (model 6E), or income level (model 6F)

Determinant	Composite Z-score for corneal nerve fibre measures											
	Model 6A		Model 6B		Model 6C		Model 6D		Model 6E		Model 6F	
	Number of participants	stβ (95% CI)	Number of participants	stβ (95% CI)	Number of participants	stβ (95% CI)	Number of participants	stβ (95% CI)	Number of participants	stβ (95% CI)	Number of participants	stβ (95% CI)
**Glucose metabolism status**	3471		3471		3160		3160		722		2706	
Prediabetes versus normal glucose metabolism	509 / 2225[a]	-0.08 (-0.18, 0.03)	509 / 2225[a]	-0.08 (-0.18, 0.02)	460 / 2035[a]	-0.10 (-0.20, 0.01)	460 / 2035[a]	-0.10 (-0.20, 0.01)	106 / 421[a]	-0.20 (-0.42, 0.02)	387 / 1754[a]	-0.07 (-0.18, 0.05)
Type-2 diabetes versus normal glucose metabolism	729 / 2225[b]	-0.14 (-0.25, -0.04)*	729 / 2225[b]	-0.15 (-0.25, -0.05)*	659 / 2035[b]	-0.16 (-0.27, -0.05)*	659 / 2035[b]	-0.17 (-0.27, -0.06)*	192 / 421[b]	-0.32 (-0.54, -0.09)*	560 / 1754[b]	-0.18 (-0.28, -0.08)*
**Measure of glycaemia**												
Fasting plasma glucose, per SD	3,470	-0.09 (-0.13, -0.05)*	3469	-0.09 (-0.12, -0.05)*	3159	-0.10 (-0.14, -0.06)*	3159	-0.10 (-0.14, -0.06)*	721	-0.09 (-0.16, -0.01)*	2705	-0.09 (-0.13, -0.04)*
2-hour post-load glucose, per SD	3328	-0.07 (-0.11, -0.03)*	3327	-0.07 (-0.11, -0.03)*	3041	-0.08 (-0.12, -0.04)*	3041	-0.08 (-0.12, -0.04)*	680	-0.12 (-0.20, -0.03)*	2595	-0.07 (-0.11, -0.02)*
$HbA1_c$, per SD	3468	-0.08 (-0.11, -0.04)*	3467	-0.08 (-0.12, -0.04)*	3157	-0.08 (-0.12, -0.04)*	3157	-0.08 (-0.12, -0.04)*	720	-0.07 (-0.15, -0.02)*	2703	-0.07 (-0.11, -0.02)*
SAF, per SD	3254	-0.05 (-0.08, -0.01)*	3253	-0.05 (-0.08, -0.01)*	2692	-0.05 (-0.09, -0.01)*	2692	-0.04 (-0.08, -0.001)*	699	-0.07 (-0.15, 0.02)*	2543	-0.05 (-0.09, -0.001)*
Duration of diabetes, per SD	574	-0.09 (-0.17, -0.001)*	574	-0.10 (-0.18, -0.01)*	515	-0.10 (-0.19, -0.01)*	515	-0.11 (-0.20, -0.02)*	160	-0.05 (-0.24, 0.14)	449	-0.07 (-0.16, 0.02)

Standardized regression coefficient (stβ) represents the differences in corneal nerve fibre measures in SD for prediabetes or type-2 diabetes versus normal glucose metabolism or per SD greater measure of glycaemia. In the glucose metabolism status, fasting plasma glucose, 2-hour post-load glucose, $HbA1_c$, SAF and duration of diabetes (only for type-2 diabetes) study populations, 1 SD corresponds with 0.9 (unit-less) for composite Z-score for corneal nerve measures, 1.4 mmol/L for fasting plasma glucose, 3.9 mmol/L for 2-hour post-load glucose, 0.8% or 8.4 mmol/mol for $HbA1_c$, 0.5 arbitrary units for SAF, and 7.4 years for the duration of diabetes. In Models 6B, 6C, 6D, 6E, 6F, values per SD were numerically comparable

Asterisks indicate values that are statistically significant ($p<0.05$)

Variables entered into the models: adjusted for corneal confocal microscopy visit interval, age, sex, educational status (where applicable), office systolic blood pressure (where applicable), total cholesterol-to-HDL cholesterol ratio, use of antihypertensive and/ or lipid-modifying medication (yes/no), waist circumference (where applicable), smoking status (never, former, current), and alcohol consumption status (none, low, high)

[a] Number of participants with prediabetes versus number of participants with normal glucose metabolism

[b] Number of participants with type-2 diabetes versus number of participants with normal glucose metabolism

ESM Table 8 Associations of glucose metabolism status and measures of glycaemia with corneal nerve fibre absolute tortuosity (model 7A), corneal nerve fibre weighted tortuosity (model 7B), and corneal nerve length divided by absolute tortuosity (model 7C)

Determinant	Number of participants	corneal nerve fibre absolute tortuosity stβ (95% CI)	Number of participants	Corneal nerve fibre weighted tortuosity stβ (95% CI)	Number of participants	Corneal nerve length / absolute tortuosity stβ (95% CI)
	Model 7A		Model 7B		Model 7C	
**Glucose metabolism status**	3471		3471		3471	
Prediabetes versus normal glucose metabolism		0.04 (-0.06, 0.14)		0.08 (-0.02, 0.18)		-0.10 (-0.20, 0.00)
Type-2 diabetes versus normal glucose metabolism		-0.04 (-0.15, 0.06)		-0.02 (-0.13, 0.08)		-0.13 (-0.23, -0.02)*
**Measure of glycaemia**						
Fasting plasma glucose, per SD	3470	-0.03 (-0.07, 0.01)	3470	-0.05 (-0.08, -0.01)*	3470	-0.07 (-0.11, -0.03)*
2-hour post-load glucose, per SD	3328	-0.02 (-0.06, 0.02)	3328	-0.02 (-0.06, 0.02)	3328	-0.06 (-0.10, -0.02)*
$HbA1_c$, per SD	3468	-0.05 (-0.09, -0.01)*	3468	-0.07 (-0.10, -0.03)*	3467	-0.05 (-0.09, -0.01)*
SAF, per SD	3254	0.01 (-0.03, 0.05)	3254	-0.003 (-0.04, 0.04)	3254	-0.05 (-0.09, -0.01)*
Duration of diabetes, per SD	574	-0.05 (-0.13, 0.04)	574	-0.04 (-0.13, 0.04)	574	-0.07 (-0.16, 0.01)

Standardized regression coefficients (stβ) represent the differences in corneal nerve fibre measures in SD for prediabetes or type-2 diabetes versus normal glucose metabolism or per SD greater measure of glycaemia. In the glucose metabolism status, fasting plasma glucose, 2-hour post-load glucose, $HbA1_c$, SAF and duration of diabetes (only for type-2 diabetes) study populations, 1 SD corresponds with 0.5 (unit-less) absolute tortuosity, 1,8 (unit-less) weighted tortuosity, 1.4 (unit-less) nerve corneal nerve length / absolute tortuosity, 1.4 mmol/L for fasting plasma glucose, 3.9 mmol/L for 2-hour post-load glucose, 0.8 % or 8.4 mmol/mol for $HbA1_c$, 0.5 arbitrary units for SAF, and 7.4 years for the duration of diabetes

Asterisks indicate values that are statistically significant ($p<0.05$)

Variables entered into the models: adjusted only for corneal confocal microscopy visit interval, age, sex, and educational status (high, medium, low), office systolic blood pressure, total cholesterol-to-HDL cholesterol ratio, use of antihypertensive and/ or lipid-modifying medication (yes/no), waist circumference, smoking status (never, former, current), and alcohol consumption status (none, low, high)

ESM Table 9 Associations of glucose metabolism status and measures of glycaemia with the composite Z-score for corneal nerve fibre measures additionally adjusted for: location of captured corneal nerve images (model 8A), the orientation of the corneal nerve fibres (model 8B), the total surface area of the captured image (model 8C), or where we included images with quality criteria of 25% or more (model 8D)

Determinant	Number of participants	Composite Z-score for corneal nerve fibre measures stβ (95% CI)	Number of participants	Composite Z-score for corneal nerve fibre measures stβ (95% CI)	Number of participants	Composite Z-score for corneal nerve fibre measures stβ (95% CI)	Number of participants	Composite Z-score for corneal nerve fibre measures stβ (95% CI)
	Model 8A		Model 8B		Model 8C		Model 8D	
**Glucose metabolism status**	3459		2149		3463		3944	
Prediabetes versus normal glucose metabolism	509 / 2223[a]	-0.07 (-0.17, 0.02)	496 / 2203[a]	-0.09 (-0.19, 0.02)	509 / 2225[a]	-0.08 (-0.17, 0.01)	581 / 2511[a]	-0.08 (-0.17, 0.02)
Type-2 diabetes versus normal glucose metabolism	727 / 2223[b]	-0.15 (-0.25, -0.05)*	701 / 2203[b]	-0.17 (-0.28, -0.07)*	729 / 2225[b]	-0.09 (-0.18, 0.01)	841 / 2511[b]	-0.13 (-0.23, -0.04)*
**Measure of glycaemia**								
Fasting plasma glucose, per SD	3466	-0.09 (-0.13, -0.05)*	3353	-0.09 (-0.13, -0.05)*	3470	-0.06 (-0.09, -0.02)*	3943	-0.07 (-0.11, -0.04)*
2-hour post-load glucose, per SD	3324	-0.07 (-0.11, -0.03)*	3213	-0.08 (-0.12, -0.04)*	3328	-0.05 (-0.09, -0.02)*	3775	-0.07 (-0.11, -0.03)*
$HbA1_c$, per SD	3464	-0.08 (-0.12, -0.04)*	3351	-0.08 (-0.12, -0.04)*	3468	-0.04 (-0.08, -0.01)*	3941	-0.06 (-0.10, -0.03)*
SAF, per SD	3250	-0.05 (-0.08, -0.01)*	3139	-0.05 (-0.09, -0.01)*	3254	-0.03 (-0.06, 0.01)	3690	-0.05 (-0.09, -0.02)*
Duration of diabetes, per SD	572	-0.10 (-0.18, -0.02)*	552	-0.09 (-0.17, 0.00)	574	-0.10 (-0.17, -0.02)*	663	-0.09 (-0.17, -0.01)*

Standardized regression coefficients (stβ) represent the differences in composite Z-score for corneal nerve fibre measures, per SD for prediabetes or type-2 diabetes versus normal glucose metabolism or per SD greater measure of glycaemia. In the glucose metabolism status, fasting plasma glucose, 2-hour post-load glucose, $HbA1_c$, SAF and duration of diabetes (only for type-2 diabetes) study populations, 1 SD corresponds with 0.9 (unit-less) for composite Z-score for corneal nerve fibre measures, 1.4 mmol/L for fasting plasma glucose, 3.9 mmol/L for 2-hour post-load glucose, 0.8% or 8.4 mmol/mol for $HbA1_c$, 0.5 arbitrary units for SAF, and 7.4 years for the duration of diabetes

Asterisks indicate values that are statistically significant ($p<0.05$)

Variables entered into the models: adjusted only for corneal confocal microscopy visit interval, age, sex, and educational status (high, medium, low), office systolic blood pressure, total cholesterol-to-HDL cholesterol ratio, use of antihypertensive and/ or lipid-modifying medication (yes/no), waist circumference, smoking status (never, former, current), and alcohol consumption status (none, low, high)

[a] Number of participants with prediabetes versus number of participants with normal glucose metabolism

[b] Number of participants with type-2 diabetes versus number of participants with normal glucose metabolism

ESM Table 10 Associations of glucose metabolism status with corneal nerve measures stratified by image location center, semi-center, or inferior whorl

Determinant	Location	Number of participants	Composite Z-score for corneal nerve fibre measures stβ (95% CI)	Corneal nerve bifurcation density stβ (95% CI)	Corneal nerve density stβ (95% CI)	Corneal nerve length stβ (95% CI)	Corneal nerve fractal dimension stβ (95% CI)	Corneal nerve weighted tortuosity stβ (95% CI)
**Glucose metabolism status**								
Prediabetes versus normal glucose metabolism	Centre	368[a]	-0.28 (-0.57, 0.01)	-0.25 (-0.54, 0.04)	-0.23 (-0.52, 0.06)	-0.26 (-0.55, 0.02)	-0.28 (-0.56, -0.01)*	-0.17 (-0.45, 0.11)
	Semi-center	2694[b]	-0.07 (-0.17, 0.06)	-0.05 (-0.17, 0.06)	-0.07 (-0.18, 0.05)	-0.06 (-0.17, 0.06)	-0.03 (-0.15, 0.08)	0.07 (-0.05, 0 .18)
	Inferior whorl	405[c]	0.04 (-0.28, 0.35)	0.05 (-0.26, 0.36)	-0.08 (-0.40, 0.23)	0.04 (-0.27, 0.35)	0.14 (-0.18, 0.45)	0.37 (0.06, 0.69)
Type-2 diabetes versus normal glucose metabolism	Centre	368[a]	-0.44 (-0.77, -0.11)*	-0.30 (-0.63, 0.04)	-0.29 (-0.63, 0.05)	-0.49 (-0.82, -0.16)*	-0.54 (-0.87, -0.22)*	-0.36 (-0.69, -0.04)*
	Semi-center	2694[b]	-0.14 (-0.25, -0.02)*	-0.12 (-0.24, -0.01)*	-0.14 (-0.26, -0.03)*	-0.10 (-0.21, -0.02)*	-0.13 (-0.25, -0.02)*	0.03 (-0.07, 0.15)
	Inferior whorl	405[c]	-0.01 (-0.31, 0.29)	0.03 (-0.27, 0.33)	-0.05 (-0.35, 0.25)	-0.01 (-0.31, 0.28)	-0.02 (-0.32, 0.28)	-0.07 (-0.37, 0.23)

Standardized regression coefficients (stβ) represent the differences in corneal nerve fibre measures in SD, per SD for prediabetes or type-2 diabetes versus normal glucose metabolism. In glucose metabolism status, study populations, centrally captured images, 1 SD corresponds with 37.8 number of branches/mm^2 for corneal nerve bifurcation density, 22.0 number of main and branches/mm^2 for corneal nerve density, 4.2 mm/mm^2 for corneal nerve length, 0.1 (unit-less) for corneal nerve fractal dimension, 2.0 for corneal nerve fibre weighted tortuosity. For the images that were captured semi-center, 1 SD corresponds with 37.6 number of branches/mm^2 for corneal nerve bifurcation density, 23.4 number of main and branches/mm^2 for corneal nerve density, 4.4 mm/mm^2 for corneal nerve length, 0.1 (unit-less) for corneal nerve fractal dimension, 1.8 for corneal nerve fibre weighted tortuosity. For the images that were captured in the inferior whorl, 1 SD corresponds with 47.5 number of branches/mm^2 for corneal nerve bifurcation density, 25.5 number of main and branches/mm^2 for corneal nerve density, 4.3 mm/mm^2 for corneal nerve length, 0.07 (unit-less) for corneal nerve fractal dimension, 1.3 for corneal nerve fibre weighted tortuosity

Asterisks indicate values that are statistically significant ($p<0.05$)

Variables entered into the models: adjusted only for corneal confocal microscopy visit interval, age, sex, and educational status (high, medium, low), office systolic blood pressure, total cholesterol-to-HDL cholesterol ratio, use of antihypertensive and/ or lipid-modifying medication (yes/no), waist circumference, smoking status (never, former, current), and alcohol consumption status (none, low, high)

[a] Number of participants with prediabetes n=68, number of participants with type-2 diabetes n=72, number of participants with other types of diabetes n=2, and number of participants with normal glucose metabolism n=226

[b] Number of participants with prediabetes n=387, number of participants with type-2 diabetes n=566, number of participants with other types of diabetes n=5, and number of participants with normal glucose metabolism n=1,736

[c] Number of participants with prediabetes n=54, number of participants with type-2 diabetes n=89, number of participants with other types of diabetes n=1, and number of participants with normal glucose metabolism n=261

ESM Table 11 Standardized regression coefficients of SAF with the composite Z-score for corneal nerve fibre measures after additional adjustment for fasting plasma glucose, 2-hour post-load glucose, or $HbA1_c$

Determinant	Composite Z-score for corneal nerve fibre measures					
	Model 3 + fasting plasma glucose		Model 3 + 2-hour post-load glucose		Model 3 + $HbA1_c$	
	Number of participants	stβ (95% CI)	Number of participants	stβ (95% CI)	Number of participants	stβ (95% CI)
SAF, per SD	3253	-0.04 (-0.08, -0.01)*	3119	-0.05 (-0.08, -0.01)*	5251	-0.04 (-0.08, -0.001)*

Standardized regression coefficients (stβ) represent the differences in composite Z-score for corneal nerve fibre measures, per SD greater index of SAF. One SD corresponds with 0.9 (unit-less) for composite Z-score for corneal nerve measures and 0.5 arbitrary units for SAF in model 3 + fasting plasma glucose. In models, 3B and 3C values, per SD were numerically comparable

Asterisks indicate values that are statistically significant ($p<0.05$)

The associations were adjusted for corneal confocal microscopy visit interval, age, sex, education level (low, middle, high), office systolic blood pressure, total cholesterol-to-HDL cholesterol ratio, use of antihypertensive and/ or lipid-modifying medication (yes/no), waist circumference, smoking status (never, former, current), and alcohol consumption status (none, low, high), and fasting plasma glucose (model 3 + fasting plasma glucose), 2-hour post-load glucose (model 3 + 2-hour post-load glucose), or $HbA1_c$ (model 3 + $HbA1_c$)

ESM Table 12 Corellations of sensory neuropathy and vibration perception with composite Z-score for corneal nerve measures in glucose metabolism strata

Determinant	Normal glucose metabolism Z-score for corneal nerve measures				Prediabetes Z-score for corneal nerve measures				Type-2 diabetes Z-score for corneal nerve measures			
	n	r	P value	95%CI	n	r	P value	95%CI	n	r	P value	95%CI
Sensory neuropathy[a]	2206	-0.04	0.07	(-0.07, 0.01)	506	-0.05	0.22	(-0.14, 0.04)	719	-0.03	0.47	(-0.11, 0.05)
Vibration perception (volt)[b]	2225	-0.05	0.03*	(-0.09, -0.03)	481	-0.11	0.01*	(-0.20, -0.03)	681	-0.12	0.003*	(-0.20, -0.03)

[a] Sensory neuropathy was defined as having neuropathic pain, impaired uni- or bilateral vibration perception, and (or) use of medication prescribed for neuropathic pain (gabapentine, pregabaline, duloxetine, amitriptyline, nortriptyline or carbamazepine, the latter only in individuals without a diagnosis of epilepsy)

[b] Vibration perception was measured three times with a Horwell Neurothesiometer (NTM) at the distal phalanx of the hallux of the right and left foot. The mean of the three NTM scores was calculated for each foot

Asterisks indicate values that are statistically significant ($p<0.05$)

ESM Table 13 Associations of age with composite Z-score for corneal nerve fibre measures

Determinant	Number of participants	Model	Composite Z-score for corneal nerve fibre measures stβ (95% CI)
Age, per SD	3471	3	-0.07 (-0.11, -0.03)*

Standardized regression coefficients (stβ) represent the differences in composite Z-score for corneal nerve fibre measures in SD, per SD for age. 1 SD corresponds with 0.9 (unit-less) for composite Z-score for corneal nerve measures and 8.7 years for age

Asterisks indicate values that are statistically significant ($p<0.05$)

Variables entered into model 3: adjusted for ‘corneal confocal microscopy visit interval’, sex, glucose metabolism status, and educational status (high, medium, low), office systolic blood pressure, total cholesterol-to-HDL cholesterol ratio, use of antihypertensive and/ or lipid-modifying medication, waist circumference, smoking status (never, former, current), and alcohol consumption status (none, low, high)

In model 3, we compared stβ of prediabetes and type-2 diabetes versus normal glucose metabolism (-0.08 SD and -0.14 SD, respectively) with stβ for age (-0.07 SD) to calculate the impact of having prediabetes and type-2 diabetes in additional years (calculated as stβ (prediabetes or type-2 diabetes) / stβ (age) * the number of years per SD). Having prediabetes corresponds approximately with approximately 10 years of aging, while having type-2 diabetes corresponds approximately with 17 years of aging
